# Independent Channel Method for Lattice Thermal Conductance in Corrugated Graphene Ribbons

**DOI:** 10.3390/nano15231811

**Published:** 2025-11-29

**Authors:** Oliver I. Barreto, Chumin Wang

**Affiliations:** Instituto de Investigaciones en Materiales, Universidad Nacional Autónoma de México, Mexico City 04510, Mexico

**Keywords:** phonon transport, Born–von Karman model, unitary transformation, zigzag-edged graphene ribbons, ripple and buckling disorders

## Abstract

Graphene’s extraordinary thermal conductivity makes it a compelling material for heat management in microelectronic circuits, lithium-ion batteries, and thermoelectric devices. In this article, we investigate its vibrational modes using a Born–von Karman model that includes first- and second-nearest-neighbor interactions. The resulting phonon dispersion relations agree well with experimental data, including acoustic flexural modes. To analyze phonon transport in mesoscopic graphene ribbons, we use both the Kubo–Greenwood and Landauer formalisms, as well as an independent channel method, which analytically maps zigzag-edged hexagonal ribbons into a set of single and dual chains via a unitary transformation. The resulting lattice thermal conductance spectra exhibit quantized steps that are smoothed in the presence of corrugations. We further explore the effects of temperature-induced rippling and buckling disorders on the phonon transport in graphene ribbons suspended over trenches. The predicted thermal conductance as a function of length and temperature closely matches experimental measurements, demonstrating the effectiveness of the independent channel method for the fully real-space modeling of corrugated graphene ribbons.

## 1. Introduction

Nowadays, semiconductor-based electronic devices are increasingly fabricated at mesoscopic scales, where thermal management constitutes a critical challenge [1]. Even modest temperature rises can severely degrade a device’s performance. In electrical insulators, heat is primarily carried by phonons, i.e., normal vibrational modes. Among emerging materials, a two-dimensional (2D) allotrope of carbon named graphene has attracted considerable attention due to its exceptional properties, including massless charge carriers [2], unusual phonon dispersion relations [3], and an ultrahigh thermal conductivity [4]. These attributes make graphene a promising candidate for advanced technological applications [5,6].

In general, the phonon transport is less studied than the electronic one, despite its importance in a wide number of physical phenomena, such as heat dissipation [7] and thermoelectricity [8]. Thermal conductivity of graphene as high as 5000 Wm^−1^ K^−1^ has been reported using Raman optothermal techniques [9], exceeding those of graphite and diamond [10]. Nevertheless, this exceptional conductivity is highly sensitive to disorder [11]. Moreover, the thermal conductivity of suspended graphene ribbons decreases with ribbon width [12] and increases with sample length for a fixed width [13].

On the theoretical side, phonon transport in graphene has been investigated using diverse approaches. For example, the Boltzmann transport equation was solved within the relaxation-time approximation, combining the effects of anharmonic interactions and edge scattering via Matthiessen’s rule [14]. Molecular dynamics simulations with Nosé–Hoover thermostats reveal almost the same thermal conductivity of graphene nanoribbons along both armchair and zigzag directions [15]. The Lanczos algorithm has also been employed to analyze the influence of edge disorder on phonon transport [16]. However, for mesoscopic graphene ribbons containing multiple structural defects, atomic-scale modeling of phonon scattering requires new strategies and approaches, because its direct calculations carried out on billions of atoms would exceed our current computational capacity.

In this article, we report a unitary transformation that maps zigzag-edged mesoscopic graphene ribbons with correlated ripple and buckling distortions onto a set of independent single and dual channels within the Born–von Karman model, including first- and second-neighbor interactions. The mathematical details of this transformation are provided in Appendix A. We have further developed a new transfer matrix method tailored for dual channels to evaluate the lattice thermal conductance within the Landauer formalism, whose results are verified by the Kubo–Greenwood formula, as shown in Appendix B. The in- and out-of-plane first- and second-neighbor restoring parameters in the Born–von Karman model are determined by fitting the measured phonon dispersion relations of graphene. Finally, the theoretical predictions of thermal conductance, including the thermal contact resistance analyzed in Appendix C, as a function of temperature and ribbon length, are compared against experimental data.

## 2. The Model

To investigate graphene’s vibrational modes, we use the Born–von Karman model, in which the interaction potentials $V_{m,n}^{\left(1\right)}$ and $V_{m,n}^{\left(2\right)}$, respectively, for first- and second-neighboring atoms *m* and *n*, with displacement vectors $u_{m}$ and $u_{n}$ from their equilibrium positions, are given by [17]

(1) $$\left\{\begin{array}{l} V_{m,n}^{\left(1\right)}=\frac{\gamma_{m,n}-\alpha_{m,n}}{2}{\left[(u_{m}-u_{n})\cdot \hat{z}\;\right]}^{\;2}+\;\frac{\alpha_{m,n}}{2}\;{\left|\;u_{m}-u_{n}\right|}^{2}\\ V_{m,n}^{\left(2\right)}=\frac{{\gamma^{\prime }}_{m,n}-{\alpha^{\prime }}_{m,n}}{2}{\left[(u_{m}-u_{n})\cdot \hat{z}\;\right]}^{\;2}+\;\frac{{\alpha^{\prime }}_{m,n}}{2}\;{\left|\;u_{m}-u_{n}\right|}^{2} \end{array}\right.,$$

where $\alpha_{m,n}$ (${\alpha^{\prime }}_{m,n}$) and $\gamma_{m,n}$ (${\gamma^{\prime }}_{m,n}$) denote the in-plane and out-of-plane restoring force constants for first (second) neighbors, respectively. In this article, we align the zigzag direction of graphene with the X-axis and include second-neighbor interactions only for bonds parallel to this axis, which capture the acoustic flexural modes (ZA) in the phonon dispersion relations of pristine graphene.

**Figure 1:**
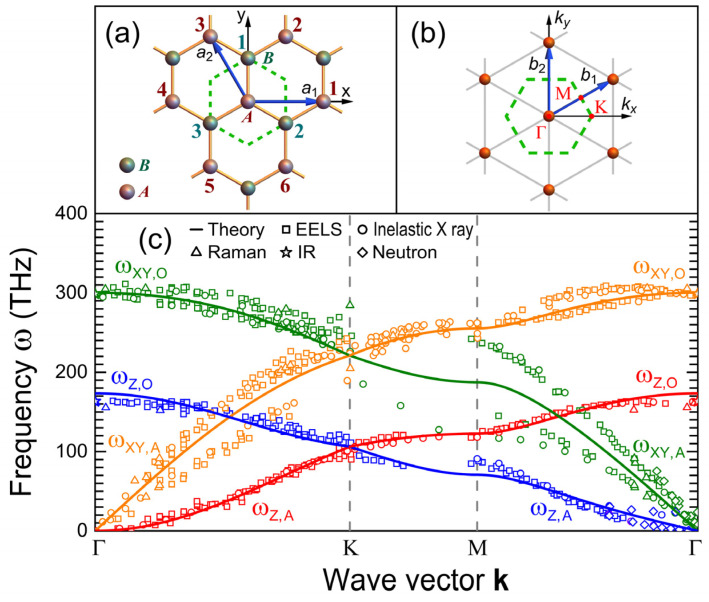
(Color online) (**a**) Portion of a graphene honeycomb lattice showing the Wigner–Seitz unit cell (dashed green lines) containing two atoms (*A* and *B*) and two primitive vectors $a_{1}$ and $a_{2}$ (blue arrows). (**b**) Corresponding reciprocal lattice (orange spheres) with the first Brillouin zone (green dashed outline) and two primitive reciprocal vectors $b_{1}$ and $b_{2}$ (blue arrows), where three high-symmetry points **Γ**, **K** and **M** are indicated by red points. (**c**) Phonon dispersion relations from the Born–von Karman model for graphene (solid lines) compared with the inelastic X-ray (circles) [18], Raman (triangles) [19], and neutron (rhombuses) [20] scatterings, as well as the infrared absorption (IR) (stars) [21] and the electron energy loss spectroscopy (EELS) (squares) [22,23,24].

Using a two-atom unit cell (see Figure 1a), the dynamical matrix $\phi_{\mu ,\nu }^{\left(s\right)}(m,n)\;=\;\partial^{2}V_{m,n}^{\left(s\right)}/\partial u_{m,\mu }\partial u_{n,\;\nu }$, with s∈ {1,2} indexing first- and second-neighbor interactions, can be written in the **k**-space as

(2) $$\Phi (k)=\left[\begin{array}{cc} 3\;\phi^{\left(1\right)}+[2-g_{2}(k)]\;\phi^{\left(2\right)} & -g_{1}(k)\;\phi^{\left(1\right)}\\ -g_{1}^{*}(k)\;\phi^{\left(1\right)} & 3\;\phi^{\left(1\right)}+[2-g_{2}(k)]\;\phi^{\left(2\right)} \end{array}\right],$$

where $g_{1}(k)=\exp(ik_{y}a_{L}/\sqrt{3})+2\exp[-ik_{y}a_{L}/\left(2\sqrt{3}\right)]\cos\;(k_{x}a_{L}/2)$, $g_{2}(k)=2\cos\;(k_{x}a_{L})$,

(3) $$\phi^{\left(1\right)}=\left[\begin{array}{ccc} \alpha & 0 & 0\\ 0 & \alpha & 0\\ 0 & 0 & \gamma \end{array}\right]\;\mathrm{and}\;\;\phi^{\left(2\right)}=\left[\begin{array}{ccc} \alpha^{\prime } & 0 & 0\\ 0 & \alpha^{\prime } & 0\\ 0 & 0 & \gamma^{\prime } \end{array}\right],$$

being $a_{L}=0.246\;\mathrm{nm}$, the lattice constant of pristine graphene. The phonon dispersion relations ω(k) follow from the secular equation $\left|\Phi (k)-M\omega^{2}(k)I\;\right|=0$, where *M* is the mass of carbon atoms, and **I** denotes the identity matrix.

High-symmetry points of the first Brillouin zone, namely Γ =(0,0), $K\;=(1\;,\;0)4\pi /\left(3a_{L}\right)$, and $M=(\sqrt{3},\;1)\pi /\left(\sqrt{3}a_{L}\right)$, are illustrated in Figure 1b. Theoretical phonon dispersion relations $\omega_{T}(k)$ (solid lines), obtained from the Born–von Karman model (Equation (1)) with the non-linear constraint for flexural modes $(\partial \omega_{\mathrm{ZA}}^{2}/\partial k_{x})(\Gamma \;)=0$ along the Γ-Κ direction, i.e., $\gamma^{\prime }=-\gamma /4$, are plotted in Figure 1c, in comparison with the experimental data obtained from the inelastic X-ray (circles) [18], Raman (triangles) [19], and neutron (rhombuses) [20] scatterings, as well as the infrared absorption (IR) (stars) [21] and the electron energy loss spectroscopy (EELS) (squares) [22,23,24].

The resulting force constants are

(4) $$\gamma \approx 104.1\;N/m,\;\mathrm{with}\;\alpha =3\gamma ,\;\alpha^{\prime }=\gamma /4\;\mathrm{and}\;\gamma^{\prime }=-\gamma /4\;,$$

obtained by minimizing the root-mean-square deviation given by

(5) $$\sigma ={\left\{\frac{1}{N}\sum_{s=1}^{N}{\left[\omega_{T}(k_{s})-\omega_{E}(k_{s})\right]}^{2}\right\}}^{1/2},$$

where N=709 is the number of experimental data $\omega_{E}(k_{s})$ of Refs. [18,19,20,21,22,23,24], shown as open symbols in Figure 1c, and they are compared with $\omega_{T}(k_{s})$ of the same color.

The analytical phonon dispersion relations, illustrated in Figure 1c, are given by

(6) $$\left\{\begin{array}{c} \omega_{\mathrm{XY},\;\Omega }^{2}(k)=\frac{\alpha \left[3\;\pm \;|g_{1}(k)|\right]\;+\alpha^{\prime }\left[2-g_{2}(k)\right]}{M}\\ \omega_{Z,\;\Omega }^{2}(k)=\frac{\gamma \left[3\;\pm \;|g_{1}(k)|\right]\;+\gamma^{\prime }\left[2-g_{2}(k)\right]}{M} \end{array}\right.,$$

where Ω∈{ O, A}. The plus (minus) sign in Equation (6) corresponds to the optical (O) [acoustic (A)] branches for both the in-plane (XY) and out-of-plane (Z) vibrational modes. As seen in Figure 1c, the theoretical dispersion relations agree well with the experimental data, capturing the essential features of the measured graphene’s phonon spectra, producing a standard deviation of σ≈15.7 THz.

**Figure 2:**
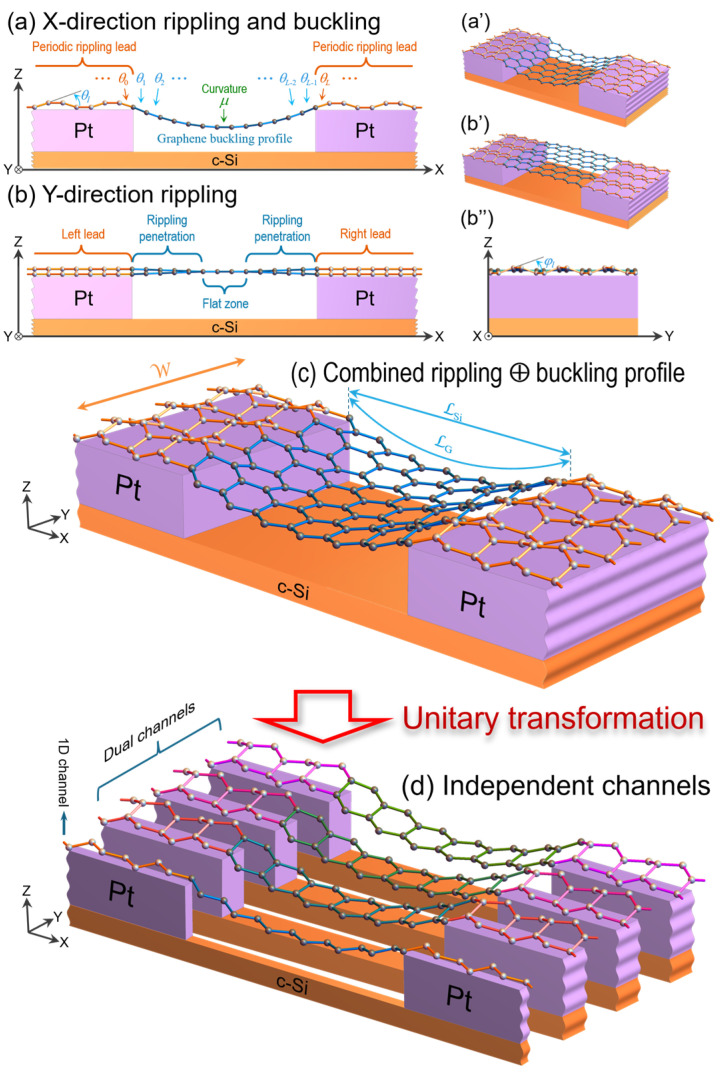
(Color online) Sketches of a zigzag-edged graphene ribbon suspended between two Pt contacts on a crystalline Si substrate. (**a**) XZ-plane side view of X-direction ripples in both leads with an angular distribution $\theta_{l}$ and a buckling profile in the suspended region. (**b**) XZ-plane side view of Y-direction ripples that gradually penetrate the suspended region; (**b’’**) YZ-plane front view illustrating the transverse angular distribution $\varphi_{l}$ in both leads. (**a’**,**b’**) 3D perspective of panels (**a**,**b**). (**c**) 3D rendering of the ribbon combining the buckling with X- and Y-direction ripples. (**d**) Independent channels obtained by applying the unitary transformation **Ξ** to the original ribbon in (**c**). Dark spheres denote atoms in the suspended ribbon, while light ones denote those in the leads.

Now, let us consider a suspended graphene ribbon of length $L_{G}$ over a trench, clamped by two metallic contacts deposited on a crystalline silicon substrate, following the experimental setup in Ref. [13]. High-temperature annealing at 573 K, followed by cooling, leads to a thermal expansion mismatch between graphene and its supporting structure that produces ripples and buckling [25] in the suspended ribbon, as schematically shown in Figure 2. These thermally induced corrugations perturb the atomic arrangement, reducing the lattice thermal conductivity. Although graphene’s ripple textures on platinum supports have been experimentally observed [26,27], their quantitative impact on phonon transport remains largely unexplored.

Figure 2 shows a suspended graphene ribbon connected to the left and right graphene leads anchored on two platinum blocks with top surface area $L_{\mathrm{Pt}}^{2}$, separated by a crystalline silicon slab of length $L_{\mathrm{Si}}$, as illustrated in Figure 2c. The thermal expansion of a material is given by [28,29]

(7) $$L_{f}=L_{i}\exp\left[\int_{T_{i}}^{T_{f}}\lambda (T)\;dT\right],$$

where λ(T) is the temperature-dependent coefficient of thermal expansion; $T_{i}$ and $T_{f}$ are the initial and final temperatures; and $L_{i}$ and $L_{f}$ are the corresponding initial and final system length. Experimental data for graphene $\lambda_{G}(T)$, platinum $\lambda_{\mathrm{Pt}}(T)$, and crystalline silicon $\lambda_{\mathrm{Si}}(T)$, are taken from Refs. [29,30,31] to determine the thermal expansion or contraction of each material.

Considering an initial temperature $T_{i}=573\;K$, at which the device has no lattice mismatch as a consequence of annealing [25], and a final measurement temperature $T_{f}\;<T_{i}$, two types of lattice-mismatch strain arise. The first occurs at the graphene–platinum interface and is described by

(8) $$\varepsilon_{G/\mathrm{Pt}}(T_{f})=\left[L_{G}^{\mathrm{Lead}}(T_{f})-L_{\mathrm{Pt}}(T_{f})\right]/L_{i}^{\mathrm{Lead}},$$

with $L_{i}^{\mathrm{Lead}}=L_{G}^{\mathrm{Lead}}(T_{i})=L_{\mathrm{Pt}}(T_{i})$. This mismatch induces ripples along both the X- and Y-directions within the graphene leads. The second mismatch strain stems from the opposite signs of the thermal expansion coefficients, $\lambda_{G}(T)<0$ and $\lambda_{\mathrm{Si}}(T)>0$, giving

(9) $$\varepsilon_{G/\mathrm{Si}}(T_{f})=\left[L_{G}(T_{f})-L_{\mathrm{Si}}(T_{f})\right]/L_{i},$$

where $L_{i}=L_{G}(T_{i})=L_{\mathrm{Si}}(T_{i})$. The strain in Equation (9) produces a buckling profile in the suspended ribbon along the X-direction, accompanied by a Y-direction rippling penetration from the left and right leads, as illustrated in Figure 2a–c. These combined corrugations are considered in this article.

Let us consider a zigzag-edged graphene ribbon that contains *L* (even number) transverse armchair lines and *W* (odd number) atoms in each line, and exhibits rippling and buckling angles $\theta_{l}$ along the X-direction (see Figure 2a) and rippling angles $\varphi_{l}$ along the Y-direction (see Figure 2b’’), as well as a rippling penetration as shown in Figure 2b. Figure 2d illustrates the mapping process of this corrugated graphene ribbon into independent single and dual channels through a unitary transformation Ξ, which block-diagonalizes the dynamical matrix **Φ** of the considered ribbon given by

(10) $$\Phi =\left[\begin{array}{ccccccc} \ddots & \ddots & \ddots & \ddots & \ddots & \vdots & ⋰\\ \ddots & A_{l-2} & B_{l-2} & C_{l-2} & 0 & 0 & \ldots \\ \ddots & B_{l-2} & A_{l-1} & B_{l-1} & C_{l-1} & 0 & \ddots \\ \ddots & C_{l-2} & B_{l-1} & A_{l} & B_{l} & C_{l} & \ddots \\ \ddots & 0 & C_{l-1} & B_{l} & A_{l+1} & B_{l+1} & \ddots \\ \ldots & 0 & 0 & C_{l} & B_{l+1} & A_{l+2} & \ddots \\ ⋰ & \vdots & \ddots & \ddots & \ddots & \ddots & \ddots \end{array}\right],$$

whose submatrices are $B_{l}=I_{W}⊗\Theta_{l}$, $C_{l}=I_{W}⊗{\Theta^{\prime }}_{l}$,

(11) $$A_{2n-1}=\left[\begin{array}{cccc} \varepsilon_{2n-1}^{-} & 0 & \ldots & 0\\ 0 & a_{2n-1} & \ddots & \vdots \\ \vdots & \ddots & \ddots & 0\\ 0 & \ldots & 0 & a_{2n-1} \end{array}\right],\;\mathrm{and}\;A_{2n}=\left[\begin{array}{cccc} a_{2n} & 0 & \ldots & 0\\ 0 & \ddots & \ddots & \vdots \\ \vdots & \ddots & a_{2n} & 0\\ 0 & \ldots & 0 & \varepsilon_{2n}^{-} \end{array}\right],$$

with 3W×3W elements each. Here, $I_{W}$ is the W×W identity matrix, ⊗ is the Kronecker product, and $\Theta_{l}$ and ${\Theta^{\prime }}_{l}$ are 3 × 3 matrices given by Equations (A22) and (A23), respectively. In Equation (11),

(12) $$\varepsilon_{l}^{-}=-\;\Theta_{l-1}-\Theta_{l}-\;{\Theta^{\prime }}_{l-2}-\;{\Theta^{\prime }}_{l}\;\mathrm{and}\;a_{l}=\left[\begin{array}{cc} {\bar{\varepsilon }}_{l} & \varphi_{l}\\ \varphi_{l} & {\bar{\varepsilon }}_{l} \end{array}\right],$$

where ${\bar{\varepsilon }}_{l}=\varepsilon_{l}^{-}-\varphi_{l}$ with $\varphi_{l}$ given by Equation (A25). The unitary transformation is given by

(13) $$\Xi =\left[\begin{array}{ccccc} \ddots & \ddots & \ddots & \vdots & ⋰\\ \ddots & U_{l-1}^{3D} & 0 & 0 & \ldots \\ \ddots & 0 & U_{l}^{3D} & 0 & \ddots \\ \ldots & 0 & 0 & U_{l+1}^{3D} & \ddots \\ ⋰ & \vdots & \ddots & \ddots & \ddots \end{array}\right],$$

whose submatrices $U_{l}^{3D}$ with 3W×3W elements can be written for l=2n−1,

(14) $$U_{2n-1}^{3D}=\frac{1}{\sqrt{W}}\left[\begin{array}{cccc} 1 & -\sqrt{2}y & \ldots & -\sqrt{2}y\\ z^{T} & R_{-}(1,1) & \ldots & R_{-}(1,N)\\ \vdots & \vdots & \ddots & \vdots \\ z^{T} & R_{-}(N,1) & \ldots & R_{-}(N,N) \end{array}\right]⊗I_{3},$$

and for l=2n,

(15) $$U_{2n}^{3D}=\frac{1}{\sqrt{W}}\left[\begin{array}{cccc} z^{T} & R_{+}(N,1) & \ldots & R_{+}(N,N)\\ \vdots & \vdots & \ddots & \vdots \\ z^{T} & R_{+}(1,1) & \ldots & R_{+}(1,N)\\ 1 & \sqrt{2}y & \ldots & \sqrt{2}y \end{array}\right]⊗I_{3},$$

where N=(W−1)/2 is the number of dual channels, y=(0,1), z=(1,1), and

(16) $$R_{\pm }(m,j)=\pm \sqrt{2}\left[\begin{array}{cc} \sin[2(2j-1)m\pi /W] & \cos[2(2j-1)m\pi /W]\\ -\sin[2(2j-1)m\pi /W] & \cos[2(2j-1)m\pi /W] \end{array}\right],$$

being m= 1,…,N and j= 1,…,N. Applying Ξ to **Φ**, i.e., $\Xi^{†}\Phi \;\Xi$, and after a reordering permutation (see, for example, Equation (A70)), we obtain a block-diagonal matrix given by

(17) $$\overset{⌢}{\Phi }=\left[\begin{array}{cccc} S_{0}^{3D} & 0 & \ldots & 0\\ 0 & D_{1}^{3D} & \ddots & \vdots \\ \vdots & \ddots & \ddots & 0\\ 0 & \ldots & 0 & D_{N}^{3D} \end{array}\right],$$

where $S_{0}^{3D}$ and $D_{j}^{3D}$ are matrices given by Equation (A34), corresponding to the single-channel and the *j*-th dual channel, respectively.

In graphene, the total thermal conductance ($K_{G}$) is the sum of the electronic ($K_{G}^{\mathrm{el}}$) and phononic $\left(K_{G}^{\mathrm{ph}}\right)$ contributions, i.e., $K_{G}=\;K_{G}^{\mathrm{el}}\;+K_{G}^{\mathrm{ph}}$. Reported values indicate $K_{G}^{\mathrm{el}}(40\;K)\;\approx \;10^{-10}W\;K^{-1}$, $K_{G}^{\mathrm{el}}(300\;K)\;\approx \;10^{-9}W\;K^{-1}$, and $K_{G}^{\mathrm{ph}}(30\;K-300\;K)\approx 10^{-7}W\;K^{-1}$ [13,32,33]; thus, $K_{G}^{\mathrm{ph}}\gg K_{G}^{\mathrm{el}}$. In particular, the phonon transport in graphene ribbons can be studied by means of the Landauer formalism [33] as

(18) $$K_{G}≅K_{G}^{\mathrm{ph}}=\frac{\hbar^{2}}{2\pi k_{B}T^{2}}\int_{0}^{\infty }d\omega \frac{\omega^{2}\exp(\hbar \omega /k_{B}T)}{{\left[\exp(\hbar \omega /k_{B}T)-1\right]}^{2}}\;T(\omega ),$$

where ℏ and $k_{B}$ are, respectively, the reduced Planck and Boltzmann constants, *T* is the temperature, and T(ω) is the phonon transmittance at angular frequency ω.

Using the independent channels in Equation (17) and the transfer matrix method for dual channels developed in Appendix B, the phonon transmittance T(ω) of a corrugated graphene ribbon, decomposed as a single channel (j=0) and *N* dual channels (see Figure 2d), can be written as

(19) $$T(\omega )=\sum_{j=0}^{N}T^{\left(j\right)}(\omega ),$$

where $T^{\left(j\right)}(\omega )$ is the phonon transmittance of the *j*-th channel given by Equation (A73).

## 3. Results

**Figure 3:**
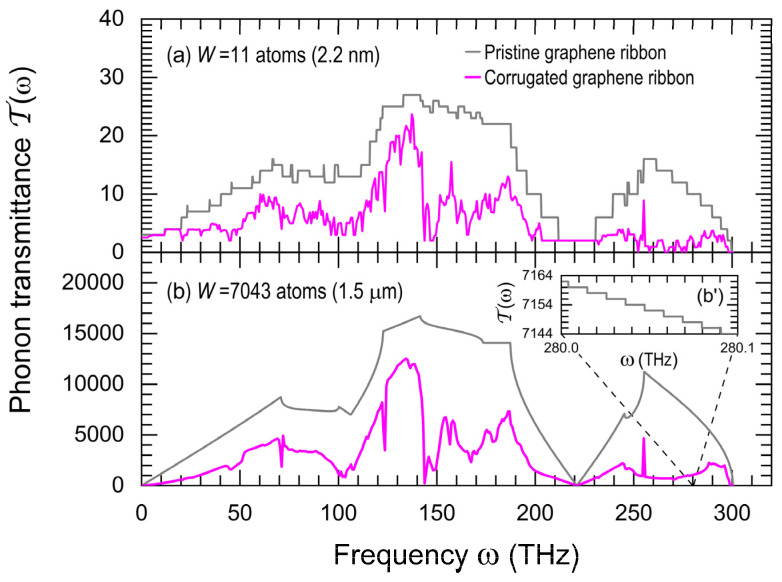
(Color online) Phonon transmittance T(ω) versus angular frequency (ω) for two graphene ribbons of length $L_{G}=0.9\;\mu m$ and width (**a**) W=11 atoms (2.2 nm) and (**b**) W=7043 atoms (1.5 μm), where gray lines represent pristine ribbons and magenta lines denote corrugated ribbons with thermal-mismatch strains at T=300 K: $\varepsilon_{G/\mathrm{Pt}}=0.00348$ in the leads yielding ripple angles $\theta_{\mathrm{lead}}\;\approx 6.76{}^{\circ}$ and $\varphi_{\mathrm{lead}}\approx 5.86{}^{\circ}$; and $\varepsilon_{G/\mathrm{Si}}=0.00185$ in the suspended region, generating the buckling profile of Equation (A16) with curvature radius $\mu^{-1}=4.33\;\mu m$. (**b’**) A magnification of (**b**).

In Figure 3, the phonon transmittance is shown for two graphene ribbons with the same length of 0.9 µm, but different widths: (a) 11 atoms and (b) 7043 atoms. The inset (b’) illustrates a magnified view of Figure 3b around ω=280 THz, where quantized values of T(ω) are observed for the pristine case. Hence, the smoothness of curves in Figure 3b is a wider ribbon effect.

In Figure 3, the phonon transmittances in pristine and corrugated graphene ribbons are, respectively, denoted by gray and magenta lines, whose graphene–platinum thermal mismatch is $\varepsilon_{G/\mathrm{Pt}}=0.00348$, obtained by assuming a flat ribbon at the annealing temperature $T_{i}=573\;K$ and a corrugated ribbon at T=300 K, which yields ripple angles $\theta_{\mathrm{lead}}\;\approx 6.76{}^{\circ}$ and $\varphi_{\mathrm{lead}}\approx 5.86{}^{\circ}$ in both graphene leads (see Equations (A15) and (A17)). A second mismatch, arising from the different thermal expansion coefficients of graphene and crystalline silicon, gives $\varepsilon_{G/\mathrm{Si}}=0.00185$. It generates a catenary-type buckling profile (see Equation (A16)) with curvature radius $\mu^{-1}=4.33\;\mu m$ at the middle of the suspended graphene ribbon, as illustrated in Figure 2a.

To compare with measured thermal conductance, the theoretical prediction must include thermal contact resistance $R_{c}(T)$ at the two interfaces between the graphene leads and the platinum electrodes, as discussed in Appendix C (see Equation (A84)). Accordingly, the predicted thermal conductance is

(20) $$K(T,L_{G})={\left[\Delta R_{G}^{\mathrm{corrugated}}(T,L_{G})+R_{G}^{\mathrm{ballistic}}(T)+R_{c}(T)\right]}^{-1},$$

where $R_{G}^{\mathrm{ballistic}}(T)$ is the ballistic thermal resistance given in Equation (A81), and $\Delta R_{G}^{\mathrm{corrugated}}(T,L_{G})=R_{G}^{\mathrm{corrugated}}(T,L_{G})-R_{G}^{\mathrm{flat}}(T,L_{G})$ accounts for the difference between the corrugated thermal resistance $R_{G}^{\mathrm{corrugated}}={\left(K_{G}^{\mathrm{corrugated}}\right)}^{-1}$ and that of a flat ribbon $R_{G}^{\mathrm{flat}}={\left(K_{G}^{\mathrm{flat}}\right)}^{-1}$, both obtained within the Born–von Karman model using Equation (18).

**Figure 4:**
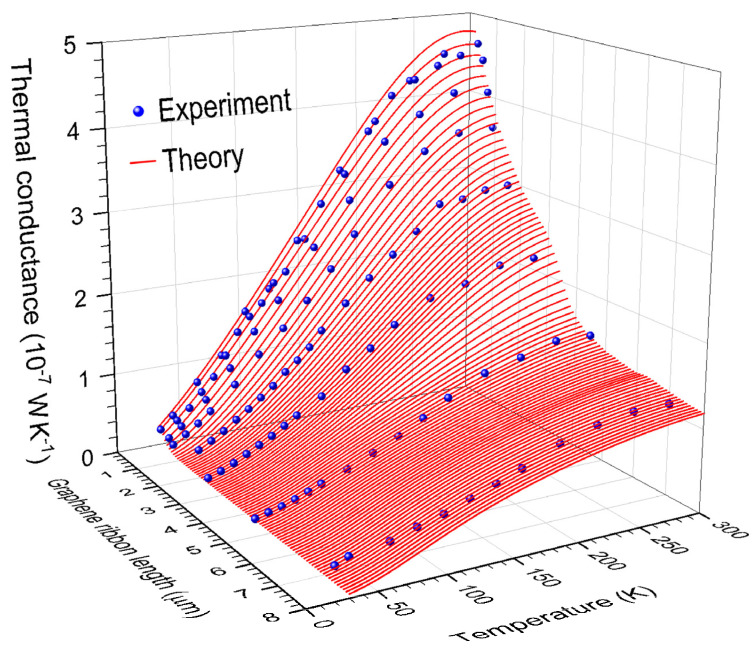
(Color online) Thermal conductance by phonons (red lines) calculated using Equation (20) as a function of temperature and ribbon length for corrugated graphene ribbons of fixed width 1.5 μm, in comparison with the experimental data (blue spheres) of Ref. [13].

In Figure 4, the predicted lattice thermal conductance (red lines) obtained from Equation (20) is compared with experimental data of Ref. [13] (blue spheres).

In Figure 4, the predicted lattice thermal conductance is obtained from Equation (20) for suspended graphene ribbons of width W=1.5 μm and a buckling profile described by Equation (A16). The ribbon is connected to two corrugated graphene leads, whose rippling angles—generated by the thermal mismatch at the graphene–platinum contact—are $\theta_{\mathrm{lead}}\;\in [6.76{}^{\circ},9.04{}^{\circ}]$ and $\varphi_{\mathrm{lead}}\in [5.86{}^{\circ},7.83{}^{\circ}]$ when the temperature decreases from 300 K to 40 K. The conductance is calculated using the independent channel method of Appendix A, together with the dual-channel transfer matrix method of Appendix B, and includes the thermal contact resistance from Appendix C. In these calculations, both the buckling profile and the ripple angles depend on the temperature, assuming a flat ribbon at 573 K immediately after annealing. We further consider the penetration of Y-oriented ripples with a depth of $L_{d}=500$ transverse lines from each lead into the suspended graphene ribbon, as illustrated in Figure 2b.

Finally, observe in Figure 4 an excellent agreement between the theoretical prediction (red lines) and the experimental data (blue spheres) from Ref. [13] over a broad range of temperatures and graphene ribbon lengths.

## 4. Conclusions

In this article, we study the normal vibrational modes and lattice thermal conductance of zigzag-edged and corrugated graphene ribbons within the Born–von Karman model. This study has been carried out by means of a new independent channel method, which maps the suspended graphene ribbon with rippling and buckling into a set of decoupled single and dual channels, using an analytical unitary transformation without introducing additional approximations (see Appendix A). We further extended the transfer matrix formalism to treat dual channels with interactions between next-nearest neighbors (see Appendix B). The in- and out-of-plane restoring force constants are determined by fitting the experimental phonon dispersion relations of pristine graphene.

To compare with experimental results, we additionally consider the thermal contact resistance and mismatches induced by differential thermal expansion, since graphene has a negative thermal expansion coefficient, while crystalline silicon and platinum have predominantly positive coefficients. In the measured device, the graphene ribbon is suspended between two platinum contacts mounted on a crystalline silicon block. The resulting expansion mismatch generates the buckled profile of the suspended section and produces ripples in the graphene leads along both the X- and Y-directions, as shown in Figure 2c.

The calculated lattice thermal conductance of corrugated graphene ribbons aligns excellently with the experimental data of Ref. [13], validating the independent channel method for mesoscopic ribbons containing over 4 × 10^8^ atoms. Beyond replicating experiments, the combination of independent channel and real-space renormalization methods establishes a robust, efficient, and scalable framework for studying the excitation dynamics in aperiodic solids [34]. Consequently, this study not only deepens our fundamental understanding of heat transport in two-dimensional materials but also paves the way for rationally designing next-generation, graphene-based thermoelectric and electronic devices, where precise thermal management is critical. Nevertheless, the present study did not include ribbon-edge reconstructions, as successfully treated in the electron transport on a graphene transistor [35]. This extension is currently under development.

## Figures and Tables

**Figure A1:**
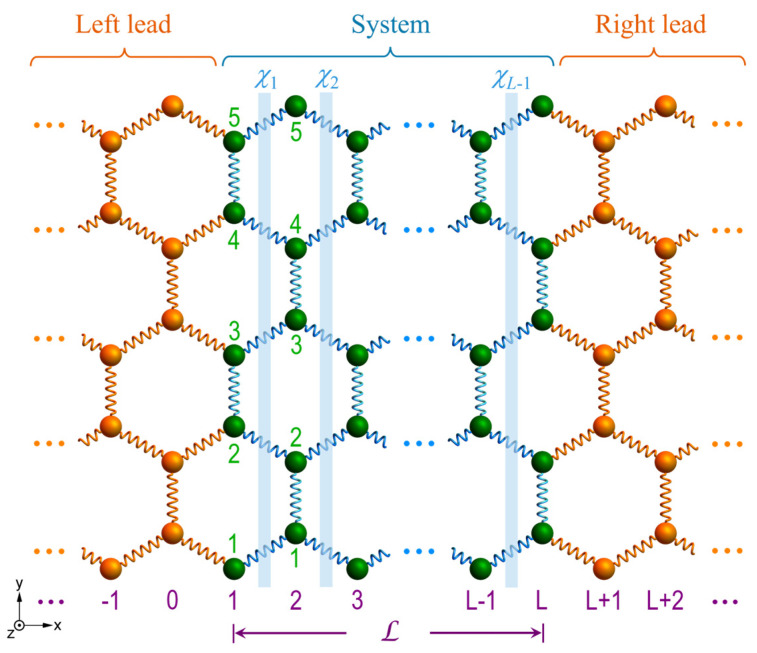
(Color online) Schematic representation of a zigzag-edged graphene ribbon (green spheres) with W=5 and an arbitrary number of transverse lines *L*, connected to two semi-infinite periodic leads (orange spheres).

**Figure A2:**
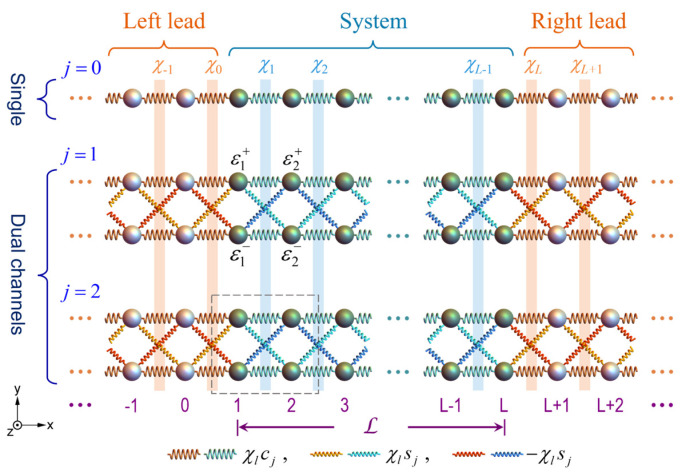
(Color online) Sketch of independent channels obtained from the zigzag-edged graphene ribbon shown in Figure A1 through the unitary transformation $\Xi_{0}$ of Equation (A5). Green spheres denote atoms in the system, while light colored ones denote those in the leads.

**Figure A3:**
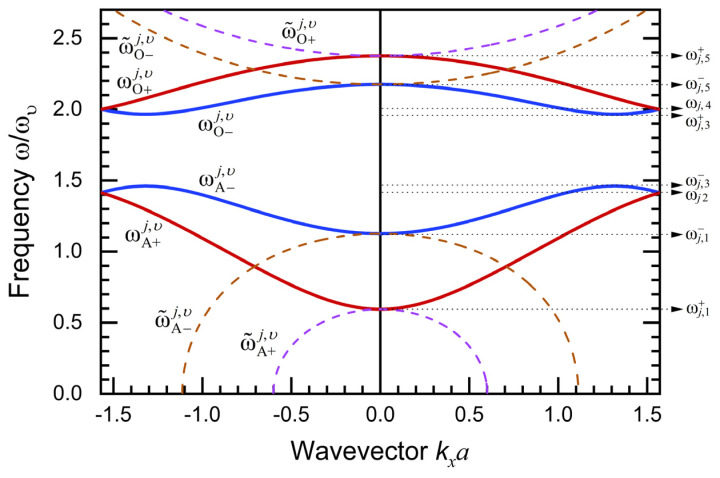
(Color online) Phonon dispersion relations $\omega_{\xi \pm }^{j,\upsilon }(k)/\omega_{\upsilon }$ (solid lines) of Equation (A50) for the *j*-th dual channel, including the evanescent modes ${\tilde{\omega }}_{\xi \pm }^{j,\upsilon }(\tilde{k})/\omega_{\upsilon }$ (dashed lines) with υ∈{x, y, z}.

**Figure A4:**
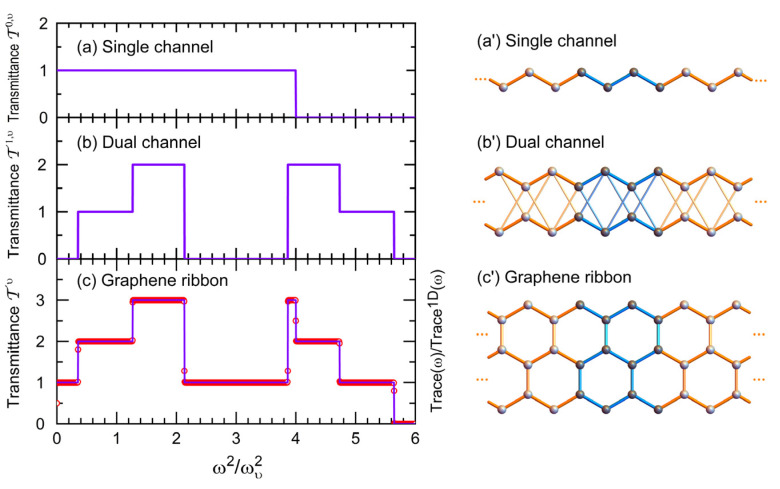
(Color online) Phonon transmittance (violet lines) of mode *υ* versus $\omega^{2}/\omega_{\upsilon }^{2}$ for (**a**) the single channel ($T^{0,\upsilon }$) sketched in (**a’**), (**b**) the dual channel ($T^{1,\upsilon }$) illustrated in (**b’**), and (**c**) the pristine graphene ribbon ($T^{\upsilon }$) drawn in (**c’**) with W=3 and L=4 (blue zone) connected to two semi-infinite leads (orange zones), in comparison with $\mathrm{Traza}(\omega )/{\mathrm{Traza}}^{1D}(\omega )$ (red circles) obtained from the Kubo–Greenwood Equation (A64) with an imaginary part of $\eta =10^{-3}\omega_{\upsilon }^{2}$ added to $\omega^{2}$.

**Figure A5:**
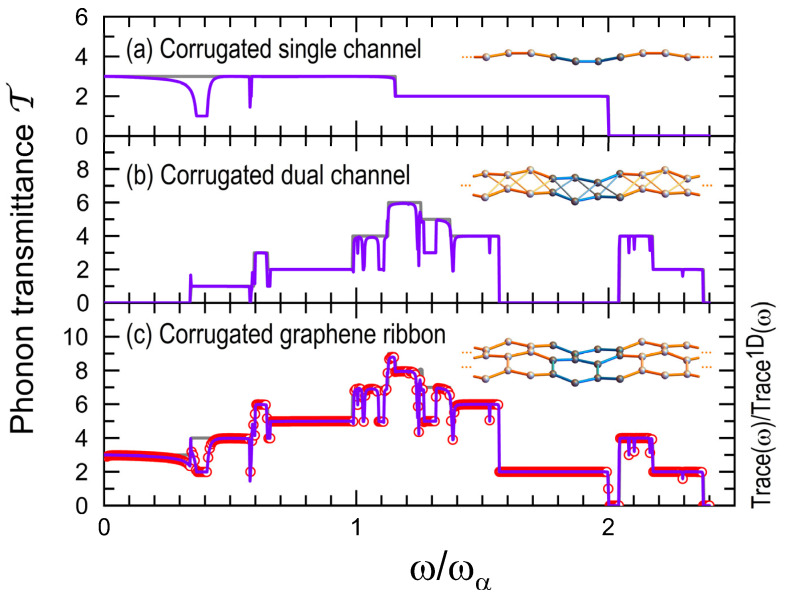
(Color online) Phonon transmittances (T) of pristine (gray lines) and corrugated (violet lines) (**a**) single channel, (**b**) dual channel, and (**c**) graphene ribbon, plotted as a function of the normalized frequency $\left(\omega /\omega_{\alpha }\right)$, obtained from the Landauer formalism and compared with the corresponding normalized trace (red circles) of the Kubo–Greenwood formula, for a corrugated graphene ribbon (blue zone) connected to two semi-infinite leads (orange zone), as illustrated in their insets.

**Figure A6:**
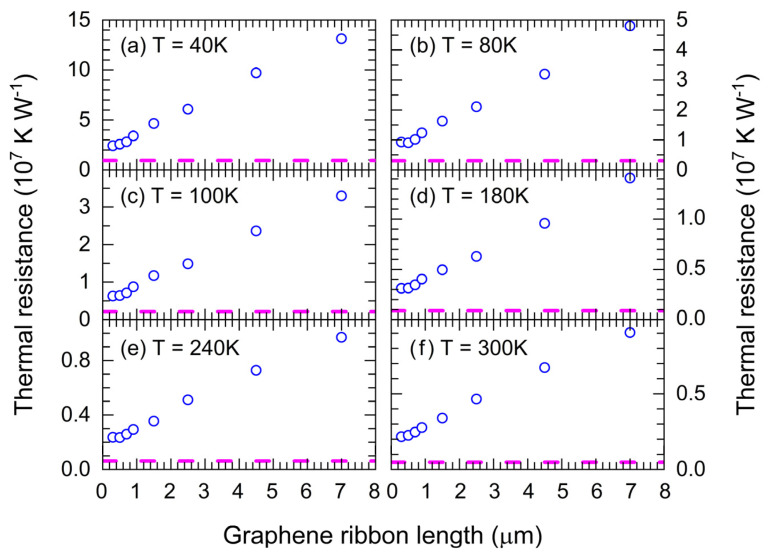
(Color online) Thermal resistance (blue open circles) extracted from the experimental data of thermal conductance reported in Ref. [13] as a function of graphene ribbon length, for temperatures of (**a**) T = 40 K, (**b**) T = 80 K, (**c**) T = 100 K, (**d**) T = 180 K, (**e**) T = 240 K, and (**f**) T = 300 K, in comparison with the ballistic thermal resistance (magenta dashed line) of pristine graphene [12].

**Figure A7:**
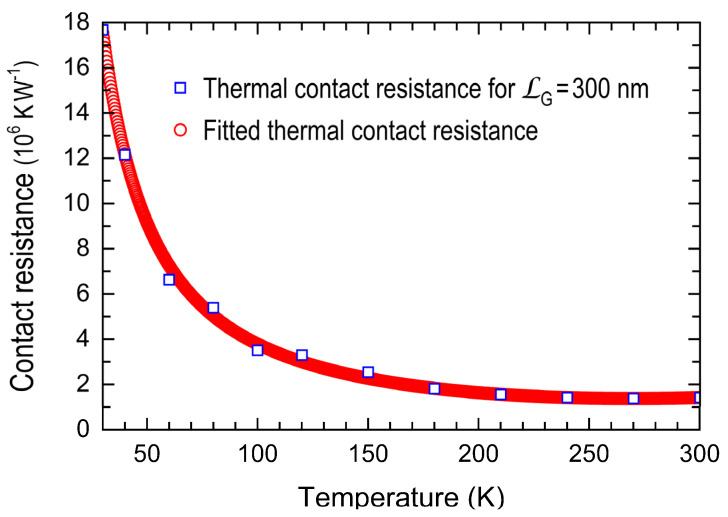
(Color online) Thermal contact resistance (blue squares) as a function of temperature, obtained from Equation (A82), in comparison with the fitted analytical expression Equation (A84) (red circles).

## Data Availability

The original contributions presented in this study are included in the article. Further inquiries can be directed to the corresponding author.

## Appendix A. Independent Channel Method

Let us consider a narrow zigzag-edged graphene ribbon of *L* transverse lines, and a width of W=5 atoms per line. This ribbon without corrugation is connected to two semi-infinite periodic leads at its longitudinal ends, as illustrated in Figure A1.

For a graphene ribbon described by a nearest-neighbor Born–von Karman model, the interaction potential of Equation (1), replacing m→(l,j) and $n\to (l^{\prime },j^{\prime })$, can be rewritten as [17]

(A1) $$V_{\left(l,j),(l^{\prime },j^{\prime }\right)}=\frac{\gamma_{l}-\alpha_{l}}{2}{\left[(u_{l,j}-u_{l^{\prime },j^{\prime }})\cdot \hat{z}\;\right]}^{\;2}+\;\frac{\alpha_{l}}{2}\;{\left|\;u_{l,j}-u_{l^{\prime },j^{\prime }}\right|}^{2},$$

where $\alpha_{l}$ and $\gamma_{l}$ are, respectively, the in-plane and out-of-plane interatomic restoring force constants along line *l* and between lines *l* and l+1, while $u_{l,j}$ is the displacement of atom (l,j) from its equilibrium position. The dynamical matrix Φ of the ribbon shown in Figure A1 is

(A2) $$\Phi =\left[\begin{array}{ccc} \Phi_{X} & 0 & 0\\ 0 & \Phi_{Y} & 0\\ 0 & 0 & \Phi_{Z} \end{array}\right],$$

where $\Phi_{X}=\Phi_{Y}=(\alpha /M)\;\Phi_{0}$ and $\Phi_{Z}=(\gamma /M)\;\Phi_{0}$ are, respectively, dynamical matrices for in-plane and out-of-plane vibrational modes with *M* the mass of carbon atoms and

(A3) $$\Phi_{0}=\left[\begin{array}{ccccc} \ddots & \ddots & \ddots & \vdots & ⋰\\ \ddots & A_{l-1} & B_{l-1} & 0\; & \ldots \\ \ddots & B_{l-1} & A_{l} & B_{l} & \ddots \\ \ldots & 0 & B_{l} & A_{l+1} & \ddots \\ ⋰ & \vdots & \ddots & \ddots & \ddots \end{array}\right],$$

where $B_{l}\;=-\chi_{l}I_{5}$ is the interaction matrix between transversal lines *l* and l+1, being $I_{n}$ the n×n identity matrix with n=5 and $\chi_{l}=\alpha_{l}/\alpha =\gamma_{l}/\gamma$. In Equation (A3), the interaction matrices within l=2m−1 (odd) and l=2m (even) numbered transverse lines, for m∈ℤ, are, respectively, in accordance with the numbering of atoms in Figure A1,

(A4) $$A_{2m-1}=\left[\begin{array}{ccccc} \varepsilon_{2m-1}^{-} & 0 & 0 & 0 & 0\\ 0 & {\bar{\varepsilon }}_{2m-1} & -1 & 0 & 0\\ 0 & -1 & {\bar{\varepsilon }}_{2m-1} & 0 & 0\\ 0 & 0 & 0 & {\bar{\varepsilon }}_{2m-1} & -1\\ 0 & 0 & 0 & -1 & {\bar{\varepsilon }}_{2m-1} \end{array}\right]\;\mathrm{and}\;A_{2m}=\left[\begin{array}{ccccc} {\bar{\varepsilon }}_{2m} & -1 & 0 & 0 & 0\\ -1 & {\bar{\varepsilon }}_{2m} & 0 & 0 & 0\\ 0 & 0 & {\bar{\varepsilon }}_{2m} & -1 & 0\\ 0 & 0 & -1 & {\bar{\varepsilon }}_{2m} & 0\\ 0 & 0 & 0 & 0 & \varepsilon_{2m}^{-} \end{array}\right],$$

where ${\bar{\varepsilon }}_{l}=\chi_{l-1}+\chi_{l}+1$ and $\varepsilon_{l}^{-}=\chi_{l-1}+\chi_{l}$.

Let us introduce a unitary transformation via matrix $\Xi_{0}$ given by

(A5) $$\Xi_{0}=\left[\begin{array}{ccccc} \ddots & \ddots & \ddots & \vdots & ⋰\\ \ddots & U_{l-1}^{\left(0\right)} & 0 & 0 & \ldots \\ \ddots & 0 & U_{l}^{\left(0\right)} & 0 & \ddots \\ \ldots & 0 & 0 & U_{l+1}^{\left(0\right)} & \ddots \\ ⋰ & \vdots & \ddots & \ddots & \ddots \end{array}\right],$$

where for the case of W=5, the submatrix $U_{l}^{\left(0\right)}$ with l=2m−1 (odd number) is

(A6) $$U_{2m-1}^{\left(0\right)}=\frac{1}{\sqrt{W}}\left[\begin{array}{ccccc} 1 & 0 & \sqrt{2} & 0 & \sqrt{2}\\ 1 & -\sqrt{2}\sin(2\vartheta_{1}) & \sqrt{2}\cos(2\vartheta_{1}) & -\sqrt{2}\sin(2\vartheta_{2}) & \sqrt{2}\cos(2\vartheta_{2})\\ 1 & \sqrt{2}\sin(2\vartheta_{1}) & \sqrt{2}\cos(2\vartheta_{1}) & \sqrt{2}\sin(2\vartheta_{2}) & \sqrt{2}\cos(2\vartheta_{2})\\ 1 & -\sqrt{2}\sin(\vartheta_{1}) & -\sqrt{2}\cos(\vartheta_{1}) & -\sqrt{2}\sin(\vartheta_{2}) & -\sqrt{2}\cos(\vartheta_{2})\\ 1 & \sqrt{2}\sin(\vartheta_{1}) & -\sqrt{2}\cos(\vartheta_{1}) & \sqrt{2}\sin(\vartheta_{2}) & -\sqrt{2}\cos(\vartheta_{2}) \end{array}\right],$$

and for l=2m (even number) is

(A7) $$U_{2m}^{\left(0\right)}=\frac{1}{\sqrt{W}}\left[\begin{array}{ccccc} 1 & -\sqrt{2}\sin(\vartheta_{1}) & -\sqrt{2}\cos(\vartheta_{1}) & -\sqrt{2}\sin(\vartheta_{2}) & -\sqrt{2}\cos(\vartheta_{2})\\ 1 & \sqrt{2}\sin(\vartheta_{1}) & -\sqrt{2}\cos(\vartheta_{1}) & \sqrt{2}\sin(\vartheta_{2}) & -\sqrt{2}\cos(\vartheta_{2})\\ 1 & -\sqrt{2}\sin(2\vartheta_{1}) & \sqrt{2}\cos(2\vartheta_{1}) & -\sqrt{2}\sin(2\vartheta_{2}) & \sqrt{2}\cos(2\vartheta_{2})\\ 1 & \sqrt{2}\sin(2\vartheta_{1}) & \sqrt{2}\cos(2\vartheta_{1}) & \sqrt{2}\sin(2\vartheta_{2}) & \sqrt{2}\cos(2\vartheta_{2})\\ 1 & 0 & \sqrt{2} & 0 & \sqrt{2} \end{array}\right],$$

being $\vartheta_{1}=\pi /5$ and $\vartheta_{2}=3\pi /5$, derived from a general expression given by

(A8) $$\vartheta_{j}=\left\{\begin{array}{l} j(\pi /W),\;\mathrm{if}\;j\;\mathrm{is}\;\mathrm{odd}\\ (W-j)(\pi /W),\;\mathrm{if}\;j\;\mathrm{is}\;\mathrm{even} \end{array}\right.,$$

with j=1,…,(W−1)/2.

Applying this unitary transformation $\Xi_{0}$ to $\Phi_{0}$ of Equation (A3), we obtain

(A9) $${\overset{⌢}{\Phi }}_{0}=\Xi_{0}^{†}\Phi_{0}\Xi_{0}=\left[\begin{array}{ccccc} \ddots & \ddots & \ddots & \vdots & ⋰\\ \ddots & {\overset{⌢}{A}}_{l-1}^{\left(0\right)} & {\overset{⌢}{B}}_{l-1}^{\left(0\right)} & 0 & \ldots \\ \ddots & {\left({\overset{⌢}{B}}_{l-1}^{\left(0\right)}\right)}^{T} & {\overset{⌢}{A}}_{l}^{\left(0\right)}\; & {\overset{⌢}{B}}_{l}^{\left(0\right)}\; & \ddots \\ \ldots & 0\; & {\left({\overset{⌢}{B}}_{l}^{\left(0\right)}\right)}^{T}\; & {\overset{⌢}{A}}_{l+1}^{\left(0\right)} & \ddots \\ ⋰ & \vdots & \ddots & \ddots & \ddots \end{array}\right],$$

where ${\left({\overset{⌢}{B}}_{l}^{\left(0\right)}\right)}^{T}$ denotes the transpose matrix of ${\overset{⌢}{B}}_{l}^{\left(0\right)}$. The transformed submatrices ${\overset{⌢}{A}}_{l}^{\left(0\right)}={\left(U_{l}^{\left(0\right)}\right)}^{†}\;A_{l}^{\left(0\right)}\;U_{l}^{\left(0\right)}$ and ${\overset{⌢}{B}}_{l}^{\left(0\right)}={\left(U_{l}^{\left(0\right)}\right)}^{†}\;B_{l}^{\left(0\right)}\;U_{l}^{\left(0\right)}$ are given by

(A10) $${\overset{⌢}{A}}_{l}^{\left(0\right)}=\left[\begin{array}{ccccc} \varepsilon_{l}^{-} & 0 & 0 & 0 & 0\\ 0 & \varepsilon_{l}^{+} & 0 & 0 & 0\\ 0 & 0 & \varepsilon_{l}^{-} & 0 & 0\\ 0 & 0 & 0 & \varepsilon_{l}^{+} & 0\\ 0 & 0 & 0 & 0 & \varepsilon_{l}^{-} \end{array}\right]\;\mathrm{and}\;{\overset{⌢}{B}}_{l}^{\left(0\right)}\;=\;-\chi_{l}\left[\begin{array}{ccccc} c_{0} & 0 & 0 & 0 & 0\\ 0 & c_{1} & {\left(-1\right)}^{l+1}s_{1} & 0 & 0\\ 0 & {\left(-1\right)}^{l}s_{1} & c_{1} & 0 & 0\\ 0 & 0 & 0 & c_{2} & {\left(-1\right)}^{l+1}s_{2}\\ 0 & 0 & 0 & {\left(-1\right)}^{l}s_{2} & c_{2} \end{array}\right],$$

where $\varepsilon_{l}^{+}=\;\chi_{l-1}+\chi_{l}+2$, $c_{0}=1$, $c_{1}=-\cos(\pi /5)$, $s_{1}=\sin(\pi /5)$, $c_{2}=\cos(2\pi /5)$, and $s_{2}=\sin(2\pi /5)$. The three colors (red, green, and blue) in Equation (A10) indicate different conducting channels: the red one corresponds to a single channel, while the green and blue ones represent two independent dual channels. Reordering the matrix ${\overset{⌢}{\Phi }}_{0}$ into block-diagonal form yields

(A11) $${\overset{⌢}{\Phi }}_{0}=\left[\begin{array}{ccc} S_{0} & 0 & 0\\ 0 & D_{1} & 0\\ 0 & 0 & D_{2} \end{array}\right],$$

where

(A12) $$S_{0}=\left[\begin{array}{ccccc} \ddots & \ddots & \ddots & \vdots & ⋰\\ \ddots & \varepsilon_{l-1}^{-} & -\chi_{l-1} & 0 & \ldots \\ \ddots & -\chi_{l-1} & \varepsilon_{l}^{-} & -\chi_{l} & \ddots \\ \ldots & 0 & -\chi_{l} & \varepsilon_{l+1}^{-} & \ddots \\ ⋰ & \vdots & \ddots & \ddots & \ddots \end{array}\right]\;\mathrm{and}\;D_{n}=\left[\begin{array}{ccccc} \ddots & \ddots & \ddots & \vdots & ⋰\\ \ddots & \varepsilon_{l-1} & d_{n,l-1} & 0 & \ldots \\ \ddots & d_{n,l-1}^{T} & \varepsilon_{l} & d_{n,l} & \ddots \\ \ldots & 0 & d_{n,l}^{T} & \varepsilon_{l+1} & \ddots \\ ⋰ & \vdots & \ddots & \ddots & \ddots \end{array}\right],$$

for n=1 or  2. In Equation (A12), $\varepsilon_{l}$ and $d_{n,l}$ are 2 × 2 matrices given by

(A13) $$\varepsilon_{l}=\left[\begin{array}{cc} \varepsilon_{l}^{+} & 0\\ 0 & \varepsilon_{l}^{-} \end{array}\right]\;\mathrm{and}\;d_{n,l}=\chi_{l}\left[\begin{array}{cc} c_{n} & {\left(-1\;\right)}^{l}s_{n}\\ {\left(-1\;\right)}^{l+1}s_{n} & c_{n} \end{array}\right].$$

It is worth mentioning that dynamical matrix (A11) can be represented by three independent channels, i.e., one single channel and two dual channels, as schematically shown in Figure A2.

In general, for an arbitrary zigzag-edged graphene ribbon with a length of *L* transverse lines and a width of *W* (odd number) atoms per line, the ribbon can be transformed into one single channel plus N= (W−1)/2 independent dual channels. The elements of matrices $d_{n,l}$ in Equation (A13), with n=1,…,N labeling the *n*-th dual channel, are given by

(A14) $$c_{n}={\left(-1\right)}^{n}\cos(n\pi /W)\;\mathrm{and}\;s_{n}=\sin(n\pi /W).$$

This independent-channel method developed for zigzag-edged graphene ribbons can be extended to include second-neighbor interactions and a buckling profile, both along the X-direction, as well as rippling distortions along the X- and Y-directions in the leads, based on a new unitary transformation given by Equation (A26). In this article, we consider two out-of-plane deformations, described below.

(1) Angles $\theta_{l}$ in the XZ subspace

The angle formed by the plane defined by two successive transverse lines *l* and *l* + 1, with respect to the X-axis, as illustrated in Figure 2a, is

(A15) $$\theta_{l}=\left\{\begin{array}{l} \theta_{\mathrm{lead}}\sin[(l-1)\pi /2],\;\;\;\;\;\;\;\;\;\;\;\;\;\;\;\;\;\;\mathrm{if}\;l<1\;\mathrm{or}\;l>L\\ \tan^{-1}[\left(y_{l+1}-y_{l}\right)/\left(x_{l+1}-x_{l}\right)],\;\mathrm{if}\;1\leq l\leq L \end{array}\right.,$$

where $\theta_{\mathrm{lead}}=\cos^{-1}(1-2\varepsilon_{G/\mathrm{Pt}})$ is the angle that generates the rippling pattern along the X direction in the left and right semi-infinite leads, being $\varepsilon_{G/\mathrm{Pt}}$ the mismatch arising from the difference between the thermal expansion coefficient of graphene $\lambda_{G}(T)<0$ and that of the platinum contacts $\lambda_{\mathrm{Pt}}(T)>0$. For example, using the thermal expansion coefficients of Refs. [30,31] and assuming an initial annealing temperature of $T_{i}=573\;K$ and a final measuring temperature of $T_{f}=300\;K$, the rippled angle in both leads $\theta_{\mathrm{lead}}\approx 6.76{}^{\circ}$ is obtained.

In Equation (A15), the suspended graphene ribbon adopts a catenary-shaped profile described by [36]

(A16) $$\left\{\begin{array}{l} x_{l}=\frac{L_{\mathrm{Si}}}{2}+\frac{1}{\mu }\sinh^{-1}\left\{\frac{\sqrt{3}}{2}(l-1)a_{G}\mu -\sinh\left(\mu L_{\mathrm{Si}}/2\right)\right\}\\ y_{l}=\frac{1}{\mu }\left\{\cosh\left[\mu \left(x_{l}-L_{\mathrm{Si}}/2\right)\right]-\cosh\left(\mu L_{\mathrm{Si}}/2\right)\right\} \end{array}\right.,$$

where $a_{G}\;=0.142\;\mathrm{nm}$ is the interatomic distance in graphene, and *μ* denotes the curvature at the lowest point $x=L_{\mathrm{Si}}/2$ of the catenary (see Figure 2a,a’), determined by $\sinh(\mu L_{\mathrm{Si}}/2)=\mu L_{G}/2$.

(2) Angles $\varphi_{l}$ in the YZ subspace

The angle between the Y-axis and the bond oriented along the Y-direction that connects neighboring atoms (*l*, *j*) and (*l*, *j* + 1), as illustrated in Figure 2b, is

(A17) $$\varphi_{l}=\left\{\begin{array}{l} (-1{)}^{l}\varphi_{\mathrm{lead}},\;\;\;\;\;\;\;\;\;\;\;\;\;\;\;\;\;\;\;\;\;\;\;\;\;\;\;\;\;\;\;\;\;\;\;\;\;\;\;\;\mathrm{if}l\leq 0\;\mathrm{or}\;l\geq L+1\\ (-1{)}^{l}[1-(l/L_{d})]\varphi_{\mathrm{lead}},\;\;\;\;\;\;\;\;\;\;\;\;\;\;\;\;\;\;\mathrm{if}\;1\leq l\leq L_{d}\\ 0,\;\;\;\;\;\;\;\;\;\;\;\;\;\;\;\;\;\;\;\;\;\;\;\;\;\;\;\;\;\;\;\;\;\;\;\;\;\;\;\;\;\;\;\;\;\;\;\;\;\;\;\;\;\;\;\;\mathrm{if}L_{d}+1\leq l\leq L-L_{d}\\ (-1{)}^{l}\{1-[(L+1-l)/L_{d}]\}\varphi_{\mathrm{lead}},\;\mathrm{if}L-L_{d}+1\leq l\leq L \end{array}\right.,$$

where $\varphi_{\mathrm{lead}}=\;\cos^{-1}(1-3\varepsilon_{G/\mathrm{Pt}}/2)$ is the out-of-plane bending angle of armchair bonds with respect to the Y-axis, and $L_{d}$ is the rippling penetration depth into the suspended ribbon inspired by experimental observations in Ref. [25], as shown in Figure 2c. Analogously to $\theta_{\mathrm{lead}}$, the graphene–platinum lattice mismatch generates $\varphi_{\mathrm{lead}}$. In this article, we use $\varphi_{\mathrm{lead}}\approx 5.86{}^{\circ}$.

From distortion angles $\theta_{l}$ and $\varphi_{l}$ given by Equations (A15) and (A17), the atomic positions are

(A18) $$r_{l,j}=r_{0,j}+H(l-1)\sum_{n=0}^{l-1}q_{n,j}-H(-l-1)\sum_{n=1}^{-l}q_{-n,j},$$

where H(x) denotes the Heaviside step function,

(A19) $$r_{0,j}\;=a\;\left(0,\;\;(j-1)\cos\varphi_{0}+\frac{1}{2}\{j-\frac{1}{2}[1+{\left(-1\right)}^{j}]\}\;,\;\;\;\frac{1}{2}[1+{\left(-1\right)}^{j}]\sin\varphi_{0}\right),$$

and

(A20) $$q_{\;l,j}=a\left(v_{x,l}\cos\theta_{l},\;\;{\left(-1\right)}^{l+j}v_{y,l,j},\;\;\;v_{x,l}\sin\theta_{l}+\frac{1}{2}{\left(-1\right)}^{j}\Delta s_{l}\;\right),$$

with $v_{x,l}\;=h_{l}\sqrt{1-h_{l}^{2}-\frac{1}{4}{\left(\Delta s_{l}\right)}^{2}}/\sqrt{h_{l}^{2}+\frac{1}{4}{\left(\Delta s_{l}\right)}^{2}\sin^{2}\theta_{l}}$, $v_{y,l,j}=\sqrt{1-\frac{1}{4}{\left(\Delta s_{l}\right)}^{2}-v_{x,l}^{2}-{\left(-1\right)}^{j}v_{x,l}\Delta s_{l}\sin\theta_{l}}$, $\Delta s_{l}=\sin|\;\varphi_{l+1}|-\sin|\;\varphi_{l}|$, and $h_{l}\;=\;\frac{1}{2}(1-\cos|\;\varphi_{l+1}|+\cos|\;\varphi_{l}|\;)-\sum_{n=1}^{l}\left(\cos|\;\varphi_{n}|-\cos|\;\varphi_{n-1}|\;\right)$.

Hence, considering the interatomic potential and the bond-angle distribution, the dynamical matrix of a graphene ribbon with buckling and rippling corrugations can be written as

(A21) $$\Phi =\left[\begin{array}{ccccccc} \ddots & \ddots & \ddots & \ddots & \ddots & \vdots & ⋰\\ \ddots & A_{l-2} & B_{l-2} & C_{l-2} & 0 & 0 & \ldots \\ \ddots & B_{l-2} & A_{l-1} & B_{l-1} & C_{l-1} & 0 & \ddots \\ \ddots & C_{l-2} & B_{l-1} & A_{l} & B_{l} & C_{l} & \ddots \\ \ddots & 0 & C_{l-1} & B_{l} & A_{l+1} & B_{l+1} & \ddots \\ \ldots & 0 & 0 & C_{l} & B_{l+1} & A_{l+2} & \ddots \\ ⋰ & \vdots & \ddots & \ddots & \ddots & \ddots & \ddots \end{array}\right],$$

where the 3W×3W submatrices $A_{l}$, $B_{l}=I_{W}⊗\Theta_{l}$ and $C_{l}=I_{W}⊗{\Theta^{\prime }}_{l}$ respectively describe intraline interactions within line *l*, interline interactions between lines *l* and l+1, and those between lines *l* and l+2, being ⊗ the Kronecker product, $I_{W}$ the W×W identity matrix,

(A22) $$\;\Theta_{l}=-\left[\begin{array}{ccc} \alpha_{l}\cos^{2}\theta_{l}+\gamma_{l}\sin^{2}\theta_{l} & 0 & (\alpha_{l}-\gamma_{l})\cos\theta_{l}\sin\theta_{l}\\ 0 & \alpha_{l} & 0\\ (\alpha_{l}-\gamma_{l})\cos\theta_{l}\sin\theta_{l} & 0 & \gamma_{l}\cos^{2}\theta_{l}+\alpha_{l}\sin^{2}\theta_{l} \end{array}\right]$$

and

(A23) $${\Theta^{\prime }}_{l}=-\left[\begin{array}{ccc} {\alpha^{\prime }}_{l}\cos^{2}{\bar{\theta }}_{l}+{\gamma^{\prime }}_{l}\sin^{2}{\bar{\theta }}_{l} & 0 & ({\alpha^{\prime }}_{l}-{\gamma^{\prime }}_{l})\cos{\bar{\theta }}_{l}\sin{\bar{\theta }}_{l}\\ 0 & \alpha_{l} & 0\\ ({\alpha^{\prime }}_{l}-{\gamma^{\prime }}_{l})\cos{\bar{\theta }}_{l}\sin{\bar{\theta }}_{l} & 0 & {\gamma^{\prime }}_{l}\cos^{2}{\bar{\theta }}_{l}+{\alpha^{\prime }}_{l}\sin^{2}{\bar{\theta }}_{l} \end{array}\right],$$

with ${\bar{\theta }}_{l}=\left(\theta_{l}+\theta_{l+1}\right)/2$. In Equation (A21), matrices $A_{l}$ for the case of W=5 are

(A24) $$A_{2n-1}=\left[\begin{array}{ccccc} \varepsilon_{2n-1}^{-} & 0 & 0 & 0 & 0\\ 0 & {\bar{\varepsilon }}_{2n-1} & \varphi_{2n-1} & 0 & 0\\ 0 & \varphi_{2n-1} & {\bar{\varepsilon }}_{2n-1} & 0 & 0\\ 0 & 0 & 0 & {\bar{\varepsilon }}_{2n-1} & \varphi_{2n-1}\\ 0 & 0 & 0 & \varphi_{2n-1} & {\bar{\varepsilon }}_{2n-1} \end{array}\right]\;\mathrm{and}\;A_{2n}=\left[\begin{array}{ccccc} {\bar{\varepsilon }}_{2n} & \varphi_{2n} & 0 & 0 & 0\\ \varphi_{2n} & {\bar{\varepsilon }}_{2n} & 0 & 0 & 0\\ 0 & 0 & {\bar{\varepsilon }}_{2n} & \varphi_{2n} & 0\\ 0 & 0 & \varphi_{2n} & {\bar{\varepsilon }}_{2n} & 0\\ 0 & 0 & 0 & 0 & \varepsilon_{2n}^{-} \end{array}\right],$$

where $\varepsilon_{l}^{-}=-\;\Theta_{l-1}-\Theta_{l}-\;{\Theta^{\prime }}_{l-2}-\;{\Theta^{\prime }}_{l}$, ${\bar{\varepsilon }}_{l}=\varepsilon_{l}^{-}-\varphi_{l}$, and

(A25) $$\varphi_{l}=-\left[\begin{array}{ccc} \alpha_{l} & 0 & 0\\ 0 & \alpha_{l}\cos^{2}\varphi_{l}+\gamma_{l}\sin^{2}\varphi_{l} & \left(\alpha_{l}-\gamma_{l}\right)\cos\varphi_{l}\sin\varphi_{l}\\ 0 & \left(\alpha_{l}-\gamma_{l}\right)\cos\varphi_{l}\sin\varphi_{l} & \gamma_{l}\cos^{2}\varphi_{l}+\alpha_{l}\sin^{2}\varphi_{l} \end{array}\right]$$

Now, let us introduce a three-dimensional (3D) unitary transformation $\Xi \;\equiv \Xi_{0}⊗I_{3}$ for vibration modes along the X-, Y- and Z-directions, where $\Xi_{0}$ is the one-dimensional (1D) unitary transformation given in Equation (A5) and $I_{3}$ is the three-dimensional identity matrix. Thus, Ξ can be written as

(A26) $$\Xi =\left[\begin{array}{ccccc} \ddots & \ddots & \ddots & \vdots & ⋰\\ \ddots & U_{l-1}^{3D} & 0 & 0 & \ldots \\ \ddots & 0 & U_{l}^{3D} & 0 & \ddots \\ \ldots & 0 & 0 & U_{l+1}^{3D} & \ddots \\ ⋰ & \vdots & \ddots & \ddots & \ddots \end{array}\right],$$

where submatrices $U_{l}^{3D}$ for W=5 and l=2n−1 (odd number) is

(A27) $$U_{2n-1}^{3D}=\frac{1}{\sqrt{W}}\left[\begin{array}{ccccc} I_{3} & 0 & \sqrt{2}I_{3} & 0 & \sqrt{2}I_{3}\\ I_{3} & -\sqrt{2}\sin(2\vartheta_{1})I_{3} & \sqrt{2}\cos(2\vartheta_{1})I_{3} & -\sqrt{2}\sin(2\vartheta_{2})I_{3} & \sqrt{2}\cos(2\vartheta_{2})I_{3}\\ I_{3} & \sqrt{2}\sin(2\vartheta_{1})I_{3} & \sqrt{2}\cos(2\vartheta_{1})I_{3} & \sqrt{2}\sin(2\vartheta_{2})I_{3} & \sqrt{2}\cos(2\vartheta_{2})I_{3}\\ I_{3} & -\sqrt{2}\sin(\vartheta_{1})I_{3} & -\sqrt{2}\cos(\vartheta_{1})I_{3} & -\sqrt{2}\sin(\vartheta_{2})I_{3} & -\sqrt{2}\cos(\vartheta_{2})I_{3}\\ I_{3} & \sqrt{2}\sin(\vartheta_{1})I_{3} & -\sqrt{2}\cos(\vartheta_{1})I_{3} & \sqrt{2}\sin(\vartheta_{2})I_{3} & -\sqrt{2}\cos(\vartheta_{2})I_{3} \end{array}\right],$$

and for W=5 and l=2n (even number) is

(A28) $$U_{2n}^{3D}=\frac{1}{\sqrt{W}}\left[\begin{array}{ccccc} I_{3} & -\sqrt{2}\sin(\vartheta_{1})I_{3} & -\sqrt{2}\cos(\vartheta_{1})I_{3} & -\sqrt{2}\sin(\vartheta_{2})I_{3} & -\sqrt{2}\cos(\vartheta_{2})I_{3}\\ I_{3} & \sqrt{2}\sin(\vartheta_{1})I_{3} & -\sqrt{2}\cos(\vartheta_{1})I_{3} & \sqrt{2}\sin(\vartheta_{2})I_{3} & -\sqrt{2}\cos(\vartheta_{2})I_{3}\\ I_{3} & -\sqrt{2}\sin(2\vartheta_{1})I_{3} & \sqrt{2}\cos(2\vartheta_{1})I_{3} & -\sqrt{2}\sin(2\vartheta_{2})I_{3} & \sqrt{2}\cos(2\vartheta_{2})I_{3}\\ I_{3} & \sqrt{2}\sin(2\vartheta_{1})I_{3} & \sqrt{2}\cos(2\vartheta_{1})I_{3} & \sqrt{2}\sin(2\vartheta_{2})I_{3} & \sqrt{2}\cos(2\vartheta_{2})I_{3}\\ I_{3} & 0 & \sqrt{2}I_{3} & 0 & \sqrt{2}I_{3} \end{array}\right],$$

with $\vartheta_{1}=\pi /5$ and $\vartheta_{2}=3\pi /5$ obtained from a general expression given by Equation (A8). Applying this unitary transformation **Ξ** to **Φ** of Equation (A21), we obtain

(A29) $$\Xi^{†}\Phi \;\Xi =\overset{⌢}{\Phi }=\left[\begin{array}{ccccccc} \ddots & \ddots & \ddots & \ddots & \ddots & \vdots & ⋰\\ \ddots & {\overset{⌢}{A}}_{l-2} & {\overset{⌢}{B}}_{l-2} & {\overset{⌢}{C}}_{l-2} & 0 & 0 & \ldots \\ \ddots & {\left({\overset{⌢}{B}}_{l-2}\right)}^{T} & {\overset{⌢}{A}}_{l-1} & {\overset{⌢}{B}}_{l-1} & {\overset{⌢}{C}}_{l-1} & 0 & \ddots \\ \ddots & {\left({\overset{⌢}{C}}_{l-2}\right)}^{T} & {\left({\overset{⌢}{B}}_{l-1}\right)}^{T} & {\overset{⌢}{A}}_{l} & {\overset{⌢}{B}}_{l} & {\overset{⌢}{C}}_{l} & \ddots \\ \ddots & 0 & {\left({\overset{⌢}{C}}_{l-1}\right)}^{T} & {\left({\overset{⌢}{B}}_{l}\right)}^{T} & {\overset{⌢}{A}}_{l+1} & {\overset{⌢}{B}}_{l+1} & \ddots \\ \ldots & 0 & 0 & {\left({\overset{⌢}{C}}_{l}\right)}^{T} & {\left({\overset{⌢}{B}}_{l+1}\right)}^{T} & {\overset{⌢}{A}}_{l+2} & \ddots \\ ⋰ & \vdots & \ddots & \ddots & \ddots & \ddots & \ddots \end{array}\right],$$

where ${\overset{⌢}{A}}_{l}={\left(U_{l}^{3D}\right)}^{†}A_{l}U_{l}^{\;\;D}$, ${\overset{⌢}{B}}_{l}={\left(U_{l}^{3D}\right)}^{†}B_{l}U_{l+1}^{\;\;D}$, and ${\overset{⌢}{C}}_{l}={\left(U_{l}^{3D}\right)}^{†}C_{l}U_{l+2}^{\;\;D}={\left(U_{l}^{3D}\right)}^{†}C_{l}U_{l}^{\;\;D}$ are given by

(A30) $${\overset{⌢}{A}}_{l}=\left[\begin{array}{ccccc} \varepsilon_{l}^{-} & 0 & 0 & 0 & 0\\ 0 & \varepsilon_{l}^{+} & 0 & 0 & 0\\ 0 & 0 & \varepsilon_{l}^{-} & 0 & 0\\ 0 & 0 & 0 & \varepsilon_{l}^{+} & 0\\ 0 & 0 & 0 & 0 & \varepsilon_{l}^{-} \end{array}\right],\;{\overset{⌢}{B}}_{l}=\;\left[\begin{array}{ccccc} c_{0} & 0 & 0 & 0 & 0\\ 0 & c_{1} & {\left(-1\right)}^{l+1}s_{1} & 0 & 0\\ 0 & {\left(-1\right)}^{l}s_{1} & c_{1} & 0 & 0\\ 0 & 0 & 0 & c_{2} & {\left(-1\right)}^{l+1}s_{2}\\ 0 & 0 & 0 & {\left(-1\right)}^{l}s_{2} & c_{2} \end{array}\right]⊗\Theta_{l}\;\mathrm{and}\;{\overset{⌢}{C}}_{l}=\left[\begin{array}{ccccc} 1 & 0 & 0 & 0 & 0\\ 0 & 1 & 0 & 0 & 0\\ 0 & 0 & 1 & 0 & 0\\ 0 & 0 & 0 & 1 & 0\\ 0 & 0 & 0 & 0 & 1 \end{array}\right]⊗{\Theta^{\prime }}_{l}$$

with $c_{j}$, $s_{j}$ defined in Equation (A14), and $\varepsilon_{l}^{+}=\varepsilon_{l}^{-}-2\varphi_{l}$. In Equation (A30), the three colors (red, green, and blue) in analogy with Equation (A11) denote the single, first-dual, and second-dual conducting channels, respectively. Reordering the dynamical matrix $\overset{⌢}{\Phi }$ into block-diagonal form yields

(A31) $$\overset{⌢}{\Phi }=\left[\begin{array}{ccc} S_{0}^{3D} & 0 & 0\\ 0 & D_{1}^{3D} & 0\\ 0 & 0 & D_{2}^{3D} \end{array}\right],$$

where $S_{0}^{3D}$ corresponds to the single channel and $D_{j}^{3D}$ to the *j*-th dual channel given by

(A32) $$S_{0}^{3D}=\left[\begin{array}{cccccc} \ddots & \ddots & \ddots & \ddots & \vdots & ⋰\\ \ddots & \varepsilon_{l-1}^{-} & \Theta_{l-1} & {\Theta^{\prime }}_{l-1} & 0 & \ldots \\ \ddots & \Theta_{l-1} & \varepsilon_{l}^{-} & \Theta_{l-2} & {\Theta^{\prime }}_{l-1} & \ddots \\ \ddots & {\Theta^{\prime }}_{l-1} & \Theta_{l-2} & \varepsilon_{l+1}^{-} & \Theta_{l-2} & \ddots \\ \ldots & 0 & {\Theta^{\prime }}_{l-1} & \Theta_{l-2} & \varepsilon_{l+2}^{-} & \ddots \\ ⋰ & \vdots & \ddots & \ddots & \ddots & \ddots \end{array}\right]\;\mathrm{and}\;D_{j}^{3D}=\left[\begin{array}{cccccc} \ddots & \ddots & \ddots & \ddots & \vdots & ⋰\\ \ddots & \varepsilon_{l-1}^{\mathrm{dual}} & \Theta_{j,l-1}^{\;\mathrm{dual}} & {\tilde{\Theta }}_{l-1}^{\;\mathrm{dual}} & 0 & \ldots \\ \ddots & {\left(\Theta_{j,l-1}^{\;\mathrm{dual}}\right)}^{T} & \varepsilon_{l}^{\mathrm{dual}} & \Theta_{j,l}^{\;\mathrm{dual}} & {\tilde{\Theta }}_{l}^{\;\mathrm{dual}} & \ddots \\ \ddots & {\tilde{\Theta }}_{l-1}^{\;\mathrm{dual}} & {\left(\Theta_{j,l}^{\;\mathrm{dual}}\right)}^{T} & \varepsilon_{l+1}^{\mathrm{dual}} & \Theta_{j,l+1}^{\;\mathrm{dual}} & \ddots \\ \ldots & 0 & {\tilde{\Theta }}_{l}^{\;\mathrm{dual}} & {\left(\Theta_{j,l+1}^{\;\mathrm{dual}}\right)}^{T} & \varepsilon_{l+2}^{\mathrm{dual}} & \ddots \\ ⋰ & \vdots & \ddots & \ddots & \ddots & \ddots \end{array}\right],$$

being $\Theta_{l}$ and ${\Theta^{\prime }}_{l}$ 3 × 3 matrices given in Equations (A22) and (A23),

(A33) $$\varepsilon_{l}^{\mathrm{dual}}=\left[\begin{array}{cc} \varepsilon_{l}^{+} & 0\\ 0 & \varepsilon_{l}^{-} \end{array}\right],\Theta_{j,l}^{\;\mathrm{dual}}=\left[\begin{array}{cc} c_{j} & {\left(-1\right)}^{l+1}s_{j}\\ {\left(-1\right)}^{l}s_{j} & c_{j} \end{array}\right]⊗\Theta_{l}\;\mathrm{and}\;{\tilde{\Theta }}_{l}^{\;\mathrm{dual}}=\left[\begin{array}{cc} 1 & 0\\ 0 & 1 \end{array}\right]⊗{\Theta^{\prime }}_{l}.$$

Redefining each pair of transverse lines as a single “new line”, Equations (A32) and (A33) can be rewritten as

(A34) $$S_{0}^{3D}=\left[\begin{array}{ccccc} \ddots & \ddots & \ddots & \vdots & ⋰\\ \ddots & S_{n-1}^{\mathrm{intra}} & S_{n-1}^{\mathrm{inter}} & 0 & \ldots \\ \ddots & {\left(S_{n-1}^{\mathrm{inter}}\right)}^{T} & S_{n}^{\mathrm{intra}} & S_{n}^{\mathrm{inter}} & \ddots \\ \ldots & 0 & {\left(S_{n}^{\mathrm{inter}}\right)}^{T} & S_{n+1}^{\mathrm{intra}} & \ddots \\ ⋰ & \vdots & \ddots & \ddots & \ddots \end{array}\right]\;\mathrm{and}\;D_{j}^{3D}=\left[\begin{array}{ccccc} \ddots & \ddots & \ddots & \vdots & ⋰\\ \ddots & D_{j,n-1}^{\mathrm{intra}} & D_{j,n-1}^{\mathrm{inter}} & 0 & \ldots \\ \ddots & {\left(D_{j,n-1}^{\mathrm{inter}}\right)}^{T} & D_{j,n}^{\mathrm{intra}} & D_{j,n}^{\mathrm{inter}} & \ddots \\ \ldots & 0 & {\left(D_{j,n}^{\mathrm{inter}}\right)}^{T} & D_{j,n+1}^{\mathrm{intra}} & \ddots \\ ⋰ & \vdots & \ddots & \ddots & \ddots \end{array}\right],$$

where n=1, 2, … , L/2 counts the new lines,

(A35) $$S_{n}^{\mathrm{intra}}=\left[\begin{array}{cc} \varepsilon_{2n-1}^{-} & \Theta_{2n-1}\\ \Theta_{2n-1} & \varepsilon_{2n}^{-} \end{array}\right],\;S_{n}^{\mathrm{inter}}=\left[\begin{array}{cc} {\Theta^{\prime }}_{2n-1} & 0\\ \Theta_{2n} & {\Theta^{\prime }}_{2n} \end{array}\right],$$

(A36) $$D_{j,n}^{\mathrm{intra}}=\left[\begin{array}{cc} \varepsilon_{2n-1}^{\mathrm{dual}} & \Theta_{j,2n-1}^{\;\mathrm{dual}}\\ \Theta_{j,2n-1}^{\;\mathrm{dual}} & \varepsilon_{2n}^{\mathrm{dual}} \end{array}\right],\;\mathrm{and}\;D_{j,n}^{\mathrm{inter}}=\left[\begin{array}{cc} {\tilde{\Theta }}_{2n-1}^{\;\mathrm{dual}} & 0\\ \Theta_{j,2n}^{\;\mathrm{dual}} & {\tilde{\Theta }}_{2n}^{\;\mathrm{dual}} \end{array}\right].$$

It is worth noting that $S_{n}^{\mathrm{intra}}$ and $S_{n}^{\mathrm{inter}}$ are 6 × 6 matrices, while $D_{j,n}^{\mathrm{intra}}$ and $D_{j,n}^{\mathrm{inter}}$ are 12 × 12 matrices.

In summary, as an example, in this Appendix we have mapped a narrow graphene ribbon of a width W=5 atoms into one single and two dual channels through unitary transformations, for both pristine and corrugated structures. In the next appendix, we resume and extend the transfer matrix method for single and dual channels, respectively.

## Appendix B. Transfer Matrices for Single and Dual Channels

For the single channel, the equation of motion derived from Equation (A12) without corrugations is

(A37) $$S_{0}u^{0,\upsilon }=(\omega^{2}/\omega_{\upsilon }^{2})\;u^{0,\upsilon },$$

where υ∈{x,y, z}, $\omega_{x}^{2}=\omega_{y}^{2}=\alpha /M$, $\omega_{z}^{2}=\gamma /M$, and $u^{0,\upsilon }={\left(\;\cdots \;,\;u_{l-1}^{0,\upsilon },\;u_{l}^{0,\upsilon },\;u_{l}^{0,\upsilon },\;\cdots \;\right)}^{T}$ is the transpose of the displacement vector, being *M* the atomic mass and α (γ) the in-plane (out-of-plane) restoring force constant from Equation (4). The transfer matrix $T_{l}^{0,\upsilon }$ derived from Equation (A37) is

(A38) $$\left[\begin{array}{c} u_{l+1}^{0,\upsilon }\\ u_{l}^{0,\upsilon } \end{array}\right]=T_{l}^{0,\upsilon }\left[\begin{array}{c} u_{l}^{0,\upsilon }\\ u_{l-1}^{0,\upsilon } \end{array}\right]=\left[\begin{array}{cc} {\left\{\varepsilon_{l}^{-}-(\omega /\omega_{\upsilon }\right)}^{2}\}\chi_{l}^{-1} & -\;\chi_{l-1}\chi_{l}^{-1}\\ 1 & 0 \end{array}\right]\left[\begin{array}{c} u_{l}^{0,\upsilon }\\ u_{l-1}^{0,\upsilon } \end{array}\right],$$

where $\varepsilon_{l}^{-}=\chi_{l-1}+\chi_{l}$. The global transfer matrix ($T_{\mathrm{sys}}^{0,\upsilon }$) connecting the left and right leads is

(A39) $$\left[\begin{array}{c} u_{L+1}^{0,\upsilon }\\ u_{L}^{0,\upsilon } \end{array}\right]=\left(\prod_{l=1}^{L}T_{L+1-l}^{0,\upsilon }\right)\left[\begin{array}{c} u_{1}^{0,\upsilon }\\ u_{0}^{0,\upsilon } \end{array}\right]=T_{\mathrm{sys}}^{0,\upsilon }\;\left[\begin{array}{c} u_{1}^{0,\upsilon }\\ u_{0}^{0,\upsilon } \end{array}\right]=\left[\begin{array}{cc} T_{1,1}^{0,\upsilon } & T_{1,2}^{0,\upsilon }\\ T_{2,1}^{0,\upsilon } & T_{2,2}^{0,\upsilon } \end{array}\right]\left[\begin{array}{c} u_{1}^{0,\upsilon }\\ u_{0}^{0,\upsilon } \end{array}\right].$$

For a vibrational wave incident from the left lead, the displacements in both semi-infinite leads are

(A40) $$u_{l}^{0,\upsilon }(k)=\left\{\begin{array}{c} e^{ikla}+r^{0,\upsilon }e^{-ikla}\;,\;\;\;\mathrm{if}l<1\;\\ t^{0,\upsilon }e^{ikla},\;\;\;\;\;\;\;\;\;\;\;\;\mathrm{if}l>L \end{array}\right.,$$

where $r^{0,\upsilon }$ and $t^{0,\upsilon }$ are, respectively, the reflection and transmission amplitudes, *k* is the wave vector, and $a=\sqrt{3}\;a_{G}/2$ with $a_{G}$ the C-C bond length in graphene. Substituting Equations (A40) into (A39) yields

(A41) $$\left[\begin{array}{c} t^{0,\upsilon }e^{ikLa}\\ 0 \end{array}\right]=Q^{-1}\left[\begin{array}{c} t^{0,\upsilon }e^{ik(L+1)a}\\ t^{0,\upsilon }e^{ikLa} \end{array}\right]=Q^{-1}\left[\begin{array}{cc} T_{1,1}^{0,\upsilon } & T_{1,2}^{0,\upsilon }\\ T_{2,1}^{0,\upsilon } & T_{2,2}^{0,\upsilon } \end{array}\right]\left[\begin{array}{c} e^{ika}+r^{0,\upsilon }e^{-ika}\\ 1+r^{0,\upsilon } \end{array}\right]=Q^{-1}\left[\begin{array}{cc} T_{1,1}^{0,\upsilon } & T_{1,2}^{0,\upsilon }\\ T_{2,1}^{0,\upsilon } & T_{2,2}^{0,\upsilon } \end{array}\right]Q\left[\begin{array}{c} 1\\ r^{0,\upsilon } \end{array}\right],$$

where Q=[w(k),w(−k)] is a 2 × 2 matrix built by column eigenvectors $w\in \{{\left(e^{ika},\;1\right)}^{T},\;{\left(-e^{ika},\;1\right)}^{T}\}$ of the single-channel *k*-space dynamical matrix $S_{0}$ for a periodic lead with $\chi_{l}=1$, given by

(A42) $$S_{0}(k)=\left[\begin{array}{cc} 2 & -1-e^{i2ka}\\ -1-e^{-i2ka} & 2 \end{array}\right].$$

Solving Equation (A41) for the reflectance ($R^{0,\upsilon }$) and transmittance ($T^{0,\upsilon }$) yields [37]

(A43) $$R^{0,\upsilon }(\omega )={\left|r^{0,\upsilon }\right|}^{2}=\frac{{\left[T_{1,1}^{0,\upsilon }-T_{2,2}^{0,\upsilon }+(T_{1,2}^{0,\upsilon }-T_{2,1}^{0,\upsilon })\cos\;(ka)\right]}^{2}+{\left(T_{1,2}^{0,\upsilon }+T_{2,1}^{0,\upsilon }\right)}^{2}\;\sin^{2}(ka)}{{\left[T_{1,2}^{0,\upsilon }-T_{2,1}^{0,\upsilon }+(T_{1,1}^{0,\upsilon }-T_{2,2}^{0,\upsilon })\cos\;(ka)\right]}^{2}+{\left(T_{1,1}^{0,\upsilon }+T_{2,2}^{0,\upsilon }\right)}^{2}\;\sin^{2}(ka)}$$

and

(A44) $$T^{0,\upsilon }(\omega )={\left|t^{0,\upsilon }\right|}^{2}=\frac{4\;\sin^{2}(ka)\;{\left[T_{1,1}^{0,\upsilon }T_{2,2}^{0,\upsilon }-T_{1,2}^{0,\upsilon }T_{2,1}^{0,\upsilon }\right]}^{2}}{{\left[T_{1,2}^{0,\upsilon }-T_{2,1}^{0,\upsilon }+(T_{1,1}^{0,\upsilon }-T_{2,2}^{0,\upsilon })\cos\;(ka)\right]}^{2}+{\left(T_{1,1}^{0,\upsilon }+T_{2,2}^{0,\upsilon }\right)}^{2}\;\sin^{2}(ka)},$$

where the phonon dispersion relation $\omega^{2}(k)=2\omega_{\upsilon }^{2}\;[1-\cos(ka)]$ has been used, with *k* the wavevector along the X-direction.

For the *j*-th dual channel, the equation of motion obtained from the matrix $D_{j}$ of Equation (A12) without corrugations reads

(A45) $$D_{j}\left[\begin{array}{c} \vdots \\ u_{l-1}^{j,\upsilon }\\ u_{l}^{j,\upsilon }\\ u_{l+1}^{j,\upsilon }\\ \vdots \end{array}\right]=\left[\begin{array}{ccccc} \ddots & \ddots & \ddots & \vdots & ⋰\\ \ddots & \varepsilon_{l-1} & d_{j,l-1} & 0 & \ldots \\ \ddots & d_{j,l-1}^{T} & \varepsilon_{l} & d_{j,l} & \ddots \\ \ldots & 0 & d_{j,l}^{T} & \varepsilon_{l+1} & \ddots \\ ⋰ & \vdots & \ddots & \ddots & \ddots \end{array}\right]\left[\begin{array}{c} \vdots \\ u_{l-1}^{j,\upsilon }\\ u_{l}^{j,\upsilon }\\ u_{l+1}^{j,\upsilon }\\ \vdots \end{array}\right]=\frac{\omega^{2}}{\omega_{\upsilon }^{2}}\left[\begin{array}{c} \vdots \\ u_{l-1}^{j,\upsilon }\\ u_{l}^{j,\upsilon }\\ u_{l+1}^{j,\upsilon }\\ \vdots \end{array}\right]$$

where $u_{l}^{j,\upsilon }=\begin{array}{cc} (u_{l,+}^{j,\upsilon }, & u_{l,-}^{j,\upsilon } \end{array}{)}^{T}$ is the displacement vector of *l*-th transverse line in the *j*-th dual channel for the vibrational mode υ∈{x,y,z}, as shown in Figure A2.

From Equation (A45) we obtain

(A46) $$u_{l+1}^{j,\upsilon }=d_{j,l}^{-1}\;[{\left(\omega /\omega_{\upsilon }\right)}^{2}-\varepsilon_{l}]\;u_{l}^{j,\upsilon }-d_{j,l}^{-1}\;d_{j,l-1}^{T}u_{l-1}^{j,\upsilon },$$

where $d_{j,l}$ describes the interaction between transverse lines *l* and *l* + 1 in the *j*-th dual channel, as given by Equation (A49). Equation (A46) can be rewritten using the 4 × 4 dual-channel transfer matrix $T_{l}^{j,\upsilon }$ as

(A47) $${\overset{⌢}{w}}_{l+1}^{j,\upsilon }\equiv \left[\begin{array}{c} u_{l+1}^{j,\upsilon }\\ u_{l}^{j,\upsilon } \end{array}\right]=\left[\begin{array}{cc} d_{j,l}^{-1}\left(I_{2}{\left(\omega /\omega_{\upsilon }\right)}^{2}-\varepsilon_{l}\right) & -\chi_{l-1}I_{2}/\chi_{l}\\ I_{2} & 0 \end{array}\right]\left[\begin{array}{c} u_{l}^{j,\upsilon }\\ u_{l-1}^{j,\upsilon } \end{array}\right]=T_{l}^{j,\upsilon }{\overset{⌢}{w}}_{l}^{j,\upsilon },$$

where $I_{2}$ is the 2 × 2 identity matrix. Thus, the total transfer matrix for the *j*-th dual channel is $M^{j,\upsilon }=\prod_{l=0}^{L+1}T_{L+1-l}^{j,\upsilon }$, which connects the left lead to the right one and satisfies

(A48) $${\overset{⌢}{w}}_{L+2}^{j,\upsilon }=\left[\begin{array}{c} u_{L+2}^{j,\upsilon }\\ u_{L+1}^{j,\upsilon } \end{array}\right]=\left(\prod_{l=0}^{L+1}T_{L+1-l}^{j,\upsilon }\right)\left[\begin{array}{c} u_{0}^{j,\upsilon }\\ u_{-1}^{j,\upsilon } \end{array}\right]=M^{j,\upsilon }{\overset{⌢}{w}}_{0}^{j,\upsilon }.$$

The normal vibrational modes in the semi-infinite leads with $\chi_{l}=1$ for l<1 or l>L can be described by a 4×1 vector $w^{j,\upsilon }(k)=\sum_{n=1}^{N_{C}}{\overset{⌢}{w}}_{2n}^{j,\upsilon }e^{ik2na}/\sqrt{N_{C}}$ with $N_{C}$ the number of unit cells in the leads, satisfying the following eigenvalue equation [38],

(A49) $$\omega_{\upsilon }^{2}\left[\begin{array}{cc} \varepsilon & (1+e^{i2ka})\;d_{j}^{T}\\ (1+e^{-i2ka})\;d_{j} & \varepsilon \end{array}\right]w_{\xi \pm }^{j,\upsilon }(k)={\left[\omega_{\xi \pm }^{j,\upsilon }(k)\right]}^{2}\;w_{\xi \pm }^{j,\upsilon }(k),$$

where the subscript ξ=A or  O denote the acoustic or optical vibrational branches, respectively. The eigenfrequencies $\omega_{\xi \pm }^{j,\upsilon }(k)$ are given by

(A50) $$\left\{\begin{array}{l} {}[\omega_{A\pm }^{j,\upsilon }(k){]}^{2}=\omega_{\upsilon }^{2}\left\{3-\sqrt{1+4\cos(ka)\;[\cos(ka)\pm \cos(j\pi /W)\;]}\right\}\\ {}[\omega_{O\pm }^{j,\upsilon }(k){]}^{2}=\omega_{\upsilon }^{2}\left\{3+\sqrt{1+4\cos(ka)\;[\cos(ka)\pm \cos(j\pi /W)]}\right\} \end{array}\right.,$$

whose corresponding eigenvectors are

(A51) $$w_{\xi \pm }^{j,\upsilon }(k)={\left(\mp {\left(-1\right)}^{j}e^{i\;ka}g_{j},\;e^{i\;ka}h_{j,\xi },\;\;g_{j},\;\;\pm {\left(-1\right)}^{j}h_{j,\xi }\right)}^{T}/\sqrt{2\;(g_{j}^{2}+h_{j,\xi }^{2})},$$

being $g_{j}=2\sin(j\pi /W)\cos(ka)$ and $h_{j,\xi \pm }=4\pm 2\cos(j\pi /W)\cos(ka)-{\left(\omega_{\xi \pm }^{j,\upsilon }/\omega_{\upsilon }\right)}^{2}$.

In Figure A3, the dispersion relations $\omega_{\xi \pm }^{j,\upsilon }(k)/\omega_{\upsilon }$ from Equation (A50) is plotted for the *j*-th dual channel, where $\omega_{j,1}^{\pm }=\omega_{\upsilon }{\left(3-c_{j}^{\pm }\right)}^{1/2}$, $\omega_{j,2}=\omega_{\upsilon }\sqrt{2}$, $\omega_{j,3}^{\pm }=\omega_{\upsilon }{\left[3\pm \sin(j\pi /W)\right]}^{1/2}$, $\omega_{j,4}=2\omega_{\upsilon }$, and $\omega_{j,5}^{\pm }=\omega_{\upsilon }{\left(3+c_{j}^{\pm }\right)}^{1/2}$, with $c_{j}^{\pm }={\left\{1+4\;[1\;\pm \cos(j\pi /W)]\right\}}^{1/2}$.

In Figure A3, observe evanescent vibrational modes in the interval $0\leq \omega \leq \omega_{j,1}^{+}$ plotted as ${\tilde{\omega }}_{A+}^{j,\upsilon }(\tilde{k})\equiv \omega_{A+}^{j,\upsilon }(ik)$ (dashed lines) possessing an imaginary wave vector $\tilde{k}=ik$ and zero transmittance. Likewise, the evanescent modes ${\tilde{w}}_{\xi \pm }^{j,\upsilon }(\tilde{k})$ in the ranges $(\omega /\omega_{\upsilon })\geq \omega_{j,5}^{-}$ and $0\leq (\omega /\omega_{\upsilon })\leq \omega_{j,1}^{-}$ are also described by Equations (A49)–(A51) substituting $\tilde{k}=ik$.

For a given frequency ω in Figure A3, there are four associated wavevectors, $k=\pm \;k_{1}$ or $\pm \;k_{2}$, which satisfy $\omega (\pm k_{1})=\omega (\pm k_{2})$, except for the forbidden frequency gap $\omega_{j,3}^{-}<(\omega /\omega_{\upsilon })<\omega_{j,3}^{+}$. For the first case that ω lies in a region with four real eigenvectors, the displacements in both leads are

(A52) $${\overset{⌢}{w}}_{2n}^{j,\upsilon }=\;\left\{\begin{array}{l} \sum_{s=1}^{2}\left\{a_{s}e^{i\;k_{s}2na}w_{\Lambda_{s}}^{j,\upsilon }(k_{s})+b_{s}e^{-i\;k_{s}2na}w_{\Lambda_{s}}^{j,\upsilon }(-k_{s})\right\},\;\mathrm{if}\;n\leq 0\\ \sum_{s=1}^{2}\{c_{s}e^{i\;k_{s}2na}w_{\Lambda_{s}}^{j,\upsilon }(k_{s})\;+d_{s}e^{-i\;k_{s}2na}w_{\Lambda_{s}}^{j,\upsilon }(-k_{s})\},\;\;\;\mathrm{if}\;n\geq \frac{L}{2}+1 \end{array}\right.,$$

where subindices $\Lambda_{s}$ denote A± or O±, depending on the frequency region of ω, $w_{\Lambda_{s}}^{j,\upsilon }(\pm k_{s})$ are given by Equation (A51), and *L* is an even integer. For an incident vibrational wave with amplitudes $a_{1}$ and $a_{2}$ coming from the left, we have $b_{s}={\overset{⌢}{r}}_{s,1}^{j,\upsilon }a_{1}+{\overset{⌢}{r}}_{s,2}^{j,\upsilon }a_{2}\;$, $c_{s}={\overset{⌢}{t}}_{s,1}^{j,\upsilon }a_{1}+{\overset{⌢}{t}}_{s,2}^{j,\upsilon }a_{2}$ and $d_{s}=0$ in Equation (A52), where ${\overset{⌢}{r}}_{s,m}^{j,\upsilon }$ and ${\overset{⌢}{t}}_{s,m}^{j,\upsilon }$ are, respectively, the reflection and transmission amplitudes of the outgoing mode $\Lambda_{s}$ from the incoming $\Lambda_{m}$.

To calculate the transmission (${\overset{⌢}{t}}^{j,\upsilon }$) and reflection (${\overset{⌢}{r}}^{j,\upsilon }$) matrices, we substitute Equation (A52) into Equation (A48), obtaining

(A53) $$\left[\begin{array}{c} {\overset{⌢}{t}}^{j,\upsilon }\\ 0_{2} \end{array}\right]=P^{j,\upsilon }\left[\begin{array}{c} I_{2}\\ {\overset{⌢}{r}}^{j,\upsilon } \end{array}\right]=\left[\begin{array}{cc} p_{+,+}^{j,\upsilon } & p_{+,-}^{j,\upsilon }\\ p_{-,+}^{j,\upsilon } & p_{-,-}^{j,\upsilon } \end{array}\right]\left[\begin{array}{c} I_{2}\\ {\overset{⌢}{r}}^{j,\upsilon } \end{array}\right],$$

where $p_{\pm ,\pm }^{j,\upsilon }$ and $p_{\pm ,\mp }^{j,\upsilon }$ are 2×2 submatrices, while $I_{2}$ and $0_{2}$ are the 2×2 identity and zero matrices. In Equation (A53), $P^{j,\upsilon }=Q^{-1}M^{j,\upsilon }Q$ with $Q=[w_{\Lambda_{1}}^{j,\upsilon }(k_{1})w_{\Lambda_{2}}^{j,\upsilon }(k_{2})w_{\Lambda_{1}}^{j,\upsilon }(-k_{1})w_{\Lambda_{2}}^{j,\upsilon }(-k_{2})\;]$, whose columns are the four eigenvectors, while

(A54) $${\overset{⌢}{r}}^{j,\upsilon }=\left[\begin{array}{cc} {\overset{⌢}{r}}_{1,1}^{j,\upsilon } & {\overset{⌢}{r}}_{1,2}^{j,\upsilon }\\ {\overset{⌢}{r}}_{2,1}^{j,\upsilon } & {\overset{⌢}{r}}_{2,2}^{j,\upsilon } \end{array}\right]\;\mathrm{and}\;{\overset{⌢}{t}}^{j,\upsilon }=\left[\begin{array}{cc} e^{ik_{1}(L+2)a}{\overset{⌢}{t}}_{1,1}^{j,\upsilon } & e^{ik_{1}(L+2)a}{\overset{⌢}{t}}_{1,2}^{j,\upsilon }\\ e^{ik_{2}(L+2)a}{\overset{⌢}{t}}_{2,1}^{j,\upsilon } & e^{ik_{2}(L+2)a}{\overset{⌢}{t}}_{2,2}^{j,\upsilon } \end{array}\right].$$

Solving Equation (A53) for ${\overset{⌢}{r}}^{j,\upsilon }$ and ${\overset{⌢}{t}}^{j,\upsilon }$ gives

(A55) $$\left\{\begin{array}{l} {\overset{⌢}{r}}^{j,\upsilon }=-(p_{-,-}^{j,\upsilon }{)}^{-1}p_{-,+}^{j,\upsilon }\\ {\overset{⌢}{t}}^{j,\upsilon }=p_{+,+}^{j,\upsilon }-p_{+,-}^{j,\upsilon }(p_{-,-}^{j,\upsilon }{)}^{-1}p_{-,+}^{j,\upsilon } \end{array}\right..$$

The flux conservation law can be written as [39]

(A56) $${\left(t^{j,\upsilon }\right)}^{†}t^{j,\upsilon }+{\left(r^{j,\upsilon }\right)}^{†}r^{j,\upsilon }=I_{2},$$

which requires normalized transmission and reflection amplitudes given by [40]

(A57) $$t_{m,s}^{j,\upsilon }=\sqrt{v_{m}/v_{s}}\;{\overset{⌢}{t}}_{m,s}^{j,\upsilon }\;andr_{m,s}^{j,\upsilon }=\sqrt{v_{m}/v_{s}}\;{\overset{⌢}{r}}_{m,s}^{j,\upsilon },$$

where $v_{s}=d\omega_{\Lambda_{s}}^{j,\upsilon }/dk_{s}$ is the group velocity of $\Lambda_{s}$ mode. Hence, the reflectance $R^{j,\upsilon }$ and transmittance $T^{j,\upsilon }$ for the *j*-th dual channel with vibration mode υ∈{x, y, z} are

(A58) $$R^{j,\upsilon }=\mathrm{Tr}[{\left(r^{j,\upsilon }\right)}^{†}r^{j,\upsilon }]\;\mathrm{and}\;T^{j,\upsilon }=\mathrm{Tr}[{\left(t^{j,\upsilon }\right)}^{†}t^{j,\upsilon }].$$

For the second case, two Bloch-type $w_{\Lambda_{1}}^{j,\upsilon }(\pm k_{1})$ and two evanescent ${\tilde{w}}_{\Lambda_{2}}^{j,\upsilon }(\pm k_{2})$ eigenvectors, Equation (A52) is replaced by

(A59) $${\overset{⌢}{w}}_{2n}^{j,\upsilon }=\;\left\{\begin{array}{l} a_{1}e^{i\;k_{1}2na}w_{\Lambda_{1}}^{j,\upsilon }(k_{1})+b_{1}e^{-i\;k_{1}2na}w_{\Lambda_{1}}^{j,\upsilon }(-k_{1})+{\tilde{a}}_{2}e^{k_{2}2na}{\tilde{w}}_{\Lambda_{2}}^{j,\upsilon }(k_{2})+{\tilde{b}}_{2}e^{-k_{2}2na}{\tilde{w}}_{\Lambda_{2}}^{j,\upsilon }(-k_{2}),\;\mathrm{if}\;n\leq 0\\ c_{1}e^{i\;k_{1}2na}w_{\Lambda_{1}}^{j,\upsilon }(k_{1})+d_{1}e^{-i\;k_{1}2na}w_{\Lambda_{1}}^{j,\upsilon }(-k_{1})+{\tilde{c}}_{2}e^{k_{2}2na}{\tilde{w}}_{\Lambda_{2}}^{j,\upsilon }(k_{2})+{\tilde{d}}_{2}e^{-k_{2}2na}{\tilde{w}}_{\Lambda_{2}}^{j,\upsilon }(-k_{2}),\;\mathrm{if}\;n\geq \frac{L+2}{2} \end{array}\right..$$

For an incident vibrational wave from the left with amplitude $a_{1}$, we set $b_{1}={\tilde{r}}^{j,\upsilon }a_{1}$, $c_{1}={\tilde{t}}^{j,\upsilon }a_{1}$ and $d_{1}=0$, where ${\tilde{r}}^{j,\upsilon }$ and ${\tilde{t}}^{j,\upsilon }$ are the transmission and reflection coefficients. To remove exponential growth terms, we choose ${\tilde{b}}_{2}={\tilde{c}}_{2}=0$. Substituting Equation (A59) into Equation (A48) yields

(A60) $$\left[\begin{array}{c} {\tilde{t}}^{j,\upsilon }e^{i\;(L+2)ak_{1}}\\ 0\\ 0\\ {\tilde{d}}_{2}e^{-(L+2)ak_{2}} \end{array}\right]={\tilde{P}}^{j,\upsilon }\left[\begin{array}{c} 1\\ {\tilde{r}}^{j,\upsilon }\\ {\tilde{a}}_{2}\\ 0 \end{array}\right]\;=\left[\begin{array}{cccc} p_{B+,B+}^{j,\upsilon } & p_{B+,B-}^{j,\upsilon } & p_{B+,E+}^{j,\upsilon } & p_{B+,E-}^{j,\upsilon }\\ p_{B-,B+}^{j,\upsilon } & p_{B-,B-}^{j,\upsilon } & p_{B-,E+}^{j,\upsilon } & p_{B-,E-}^{j,\upsilon }\\ p_{E+,B+}^{j,\upsilon } & p_{E+,B-}^{j,\upsilon } & p_{E+,E+}^{j,\upsilon } & p_{E+,E-}^{j,\upsilon }\\ p_{E-,B+}^{j,\upsilon } & p_{E-,B-}^{j,\upsilon } & p_{E-,E+}^{j,\upsilon } & p_{E-,E-}^{j,\upsilon } \end{array}\right]\left[\begin{array}{c} 1\\ {\tilde{r}}^{j,\upsilon }\\ {\tilde{a}}_{2}\\ 0 \end{array}\right],$$

where ${\tilde{P}}^{j,\upsilon }={\tilde{Q}}^{-1}M^{j,\upsilon }\tilde{Q}$ with $\tilde{Q}=[w_{\Lambda_{1}}^{j,\upsilon }(k_{1})w_{\Lambda_{1}}^{j,\upsilon }(-k_{1}){\tilde{w}}_{\Lambda_{2}}^{j,\upsilon }(k_{2}){\tilde{w}}_{\Lambda_{2}}^{j,\upsilon }(-k_{2})\;]$; subscripts B and E label Bloch or evanescent modes, respectively. The four coupled Equation (A60) determine ${\tilde{t}}^{j,\upsilon }$, ${\tilde{r}}^{j,\upsilon }$, ${\tilde{a}}_{2}$ and ${\tilde{d}}_{2}$. Using the substitution method, ${\tilde{a}}_{2}$ and ${\tilde{d}}_{2}$ can be removed, giving

(A61) $$\left[\begin{array}{c} {\tilde{t}}^{j,\upsilon }e^{i\;(L+2)ak_{1}}\\ 0 \end{array}\right]\;=\;\left[\begin{array}{cc} {\tilde{p}}_{B+,B+}^{j,\upsilon } & {\tilde{p}}_{B+,B-}^{j,\upsilon }\\ {\tilde{p}}_{B-,B+}^{j,\upsilon } & {\tilde{p}}_{B-,B-}^{j,\upsilon } \end{array}\right]\left[\begin{array}{c} 1\\ {\tilde{r}}^{j,\upsilon } \end{array}\right],$$

where ${\tilde{p}}_{\mu ,\nu }^{j,\upsilon }\;\equiv \;p_{\mu ,\nu }^{j,\upsilon }-p_{\mu ,E+}^{j,\upsilon }{\left(p_{E+,E+}^{j,\upsilon }\right)}^{-1}p_{E+,\nu }^{j,\upsilon }$ with $\mu ,\nu \in \left\{B+,\;B-\right\}$. Solving Equation (A61) we find

(A62) $$\left\{\begin{array}{l} {\tilde{r}}^{j,\upsilon }=-({\tilde{p}}_{B-,B-}^{j,\upsilon }{)}^{-1}{\tilde{p}}_{B-,B+}^{j,\upsilon }\\ {\tilde{t}}^{j,\upsilon }=e^{-i\;k_{1}(L+2)a}[{\tilde{p}}_{B+,B+}^{j,\upsilon }-{\tilde{p}}_{B+,B-}^{j,\upsilon }\;({\tilde{p}}_{B-,B-}^{j,\upsilon }{)}^{-1}{\tilde{p}}_{B-,B+}^{j,\upsilon }] \end{array}\right.,$$

which yields the reflectance $R^{j,\upsilon }=\;|{\tilde{r}}^{j,\upsilon }{|}^{2}$ and transmittance $T^{j,\upsilon }=\;|{\tilde{t}}^{j,\upsilon }{|}^{2}$.

Hence, combining the single-channel transmittance of Equation (A44) with the dual-channel ones of Equations (A58) or (A62), the total transmittance $T^{\upsilon }(\omega )$ for the mode υ∈{x, y, z} is

(A63) $$T^{\upsilon }(\omega )=\sum_{j=0}^{N}T^{j,\upsilon }(\omega ),$$

where N=(W−1)/2 is the number of dual channels and j=0 labels the single channel.

As an example, let us consider a small pristine zigzag-edged graphene ribbon (blue zone) of width W =3 and length L =4, connected to two semi-infinite leads (orange zone), as sketched in Figure A4c’. After applying the unitary transformation (A5) to the system dynamical matrix, which becomes block diagonal, consisting of a single channel (see Figure A4a’) and a dual channel (see Figure A4b’), whose phonon transmittances (violet lines) versus $\omega^{2}/\omega_{\upsilon }^{2}$ are, respectively, presented in Figure A4a $T^{\;0,\upsilon }$ and Figure A4b $T^{\;1,\upsilon }$, where υ∈{x, y, z}. The total phonon transmittance $\left(T^{\;\upsilon }=T^{\;0,\upsilon }+T^{\;1,\upsilon }\right)$ is plotted in Figure A4c and compared with the normalized trace (red circles) obtained from the Kubo–Greenwood formula given by [37,41]

(A64) TrAυIm[G(ω2/ωυ2)] AυIm[G(ω2/ωυ2)]/Traza1D(ω2/ωυ2)

where ${\mathrm{Traza}}^{1D}(\omega^{2}/\omega_{\upsilon }^{2})=-{\left(L-1\right)}^{2}a^{2}/8$, $A^{\upsilon }(l,j)=\Phi_{0}(l,j)(r_{l,\upsilon }-r_{j,\upsilon })/2$, **G** is the phonon Green’s function determined by the Dyson equation $\{M(\omega^{2}/\omega_{\upsilon }^{2})I-\Phi_{0}\}G=I$, with $\Phi_{0}$ given in Equation (A3). In Figure A4c, the normalized trace of Equation (A64) was calculated without using the independent channel method, where a small imaginary part $\eta =10^{-3}\omega_{\upsilon }^{2}$ was added to $\omega^{2}$, and two periodic graphene leads containing 15,000 atoms in each one were used.

Moreover, the spectral gap in Figure A4b arises from two sites per transverse section in the dual channel, which produces an acoustic and an optical branch. Note the spectral symmetry about $\omega^{2}=3\;\omega_{\upsilon }^{2}$ in Figure A4b), which is broken in the total transmittance spectrum shown in Figure A4c due to the different coordination numbers of edge (two nearest neighbors) and interior atoms (three nearest neighbors) in graphene ribbons.

When buckling and rippling corrugations are introduced into the small graphene ribbon illustrated in Figure A4c’, with W=3 atoms and L=4 suspended transverse lines, the new atomic positions in Cartesian coordinates X, Y and Z are, respectively, listed in Table A1, Table A2 and Table A3 in the units of $a_{G}=0.142\;nm$, including two transverse lines of the left (l=0, −1) and right (l=5, 6) leads. The buckling profile of the system (3 × 4 atoms) is characterized by $\theta_{1}$, the X-direction rippling corrugation in both leads by $\theta_{0}$, and the Y-direction rippling in the ribbon and leads by $\varphi_{0}$.

**Table A1:** X-coordinates of carbon atoms in a suspended zigzag-edged graphene ribbon (light-blue highlighted zone) connected to corrugated leads represented by l=0, −1 and l=5, 6.

In Units of $a_{G}$	j = 1	j = 2	j = 3
⁝	⁝	⁝	⁝
l=−1	$-\sqrt{3}(\;1+\cos\theta_{0})/2$	$-\sqrt{3}(\;1+\cos\theta_{0})/2$	$-\sqrt{3}(\;1+\cos\theta_{0})/2$
l=0	$-\sqrt{3}(\cos\theta_{0})/2$	$-\sqrt{3}(\cos\theta_{0})/2$	$-\sqrt{3}(\cos\theta_{0})/2$
l=1	0	0	0
l=2	$\sqrt{3}\left(\cos\theta_{1}\right)/2$	$\sqrt{3}\left(\cos\theta_{1}\right)/2$	$\sqrt{3}\left(\cos\theta_{1}\right)/2$
l=3	$\sqrt{3}(\;1+\cos\theta_{1})/2$	$\sqrt{3}(\;1+\cos\theta_{1})/2$	$\sqrt{3}(\;1+\cos\theta_{1})/2$
l=4	$\sqrt{3}(\;1+2\cos\theta_{1})/2$	$\sqrt{3}(\;1+2\cos\theta_{1})/2$	$\sqrt{3}(\;1+2\cos\theta_{1})/2$
l=5	$\sqrt{3}(\;1+2\cos\theta_{1}+\cos\theta_{0})/2$	$\sqrt{3}(\;1+2\cos\theta_{1}+\cos\theta_{0})/2$	$\sqrt{3}(\;1+2\cos\theta_{1}+\cos\theta_{0})/2$
l=6	$\sqrt{3}(\;2+2\cos\theta_{1}+\cos\theta_{0})/2$	$\sqrt{3}(\;2+2\cos\theta_{1}+\cos\theta_{0})/2$	$\sqrt{3}(\;2+2\cos\theta_{1}+\cos\theta_{0})/2$
⁝	⁝	⁝	⁝

The three vertical dots ‘⁝’ mean that a sequence continues infinitely.

**Table A2:** Y-coordinates of carbon atoms in a suspended zigzag-edged graphene ribbon (light-blue highlighted zone) connected to corrugated leads represented by l=0, −1 and l=5, 6.

$\mathrm{In}\;\mathrm{Units}\;\mathrm{of}\;a_{G}$	j = 1	j = 2	j = 3
⁝	⁝	⁝	⁝
l=−1	1/2	$1/2+\cos\varphi_{0}$	$3/2+2\cos\varphi_{0}$
l=0	0	$1+\cos\varphi_{0}$	$1+2\cos\varphi_{0}$
l=1	1/2	$1/2+\cos\varphi_{0}$	$3/2+2\cos\varphi_{0}$
l=2	0	$1+\cos\varphi_{0}$	$1+2\cos\varphi_{0}$
l=3	1/2	$1/2+\cos\varphi_{0}$	$3/2+2\cos\varphi_{0}$
l=4	0	$1+\cos\varphi_{0}$	$1+2\cos\varphi_{0}$
l=5	1/2	$1/2+\cos\varphi_{0}$	$3/2+2\cos\varphi_{0}$
l=6	0	$1+\cos\varphi_{0}$	$1+2\cos\varphi_{0}$
⁝	⁝	⁝	⁝

The three vertical dots ‘⁝’ mean that a sequence continues infinitely.

**Table A3:** Z-coordinates of carbon atoms in a suspended zigzag-edged graphene ribbon (light-blue highlighted zone) connected to corrugated leads represented by l=0, −1 and l=5, 6.

$\mathrm{In}\;\mathrm{Units}\;\mathrm{of}\;a_{G}$	j = 1	j = 2	j = 3
⁝	⁝	⁝	⁝
l=−1	$\sqrt{3}\sin\theta_{0}/2$	$\sin\varphi_{0}+\sqrt{3}\;\sin\theta_{0}/2$	$\sqrt{3}\sin\theta_{0}/2$
l=0	$\sqrt{3}\sin\theta_{0}/2$	$\sin\varphi_{0}+\sqrt{3}\;\sin\theta_{0}/2$	$\sqrt{3}\sin\theta_{0}/2$
l=1	0	$\sin\varphi_{0}$	0
l=2	$-\sqrt{3}\sin\theta_{1}/2$	$\sin\varphi_{0}-\sqrt{3}\sin\theta_{1}/2$	$-\sqrt{3}\sin\theta_{1}/2$
l=3	$-\sqrt{3}\sin\theta_{1}/2$	$\sin\varphi_{0}-\sqrt{3}\sin\theta_{1}/2$	$-\sqrt{3}\sin\theta_{1}/2$
l=4	0	$\sin\varphi_{0}$	0
l=5	$\sqrt{3}\sin\theta_{0}/2$	$\sin\varphi_{0}+\sqrt{3}\;\sin\theta_{0}/2$	$\sqrt{3}\sin\theta_{0}/2$
l=6	$\sqrt{3}\sin\theta_{0}/2$	$\sin\varphi_{0}+\sqrt{3}\;\sin\theta_{0}/2$	$\sqrt{3}\sin\theta_{0}/2$
⁝	⁝	⁝	⁝

The three vertical dots ‘⁝’ mean that a sequence continues infinitely.

To illustrate the independent channel method, let us consider an isolated, small zigzag-edged graphene ribbon of 3 × 4 atoms, with buckling and rippling corrugations as specified in Table A1, Table A2 and Table A3. Using Equations (4) and (A22)–(A26) with $\alpha_{l}\;=\alpha =3\gamma =3\gamma_{l}$, ${\alpha^{\prime }}_{l}\;=\gamma /4$ and ${\gamma^{\prime }}_{l}\;=-\gamma /4$, the dynamical matrix of this ribbon without connecting to the leads is

(A65) $$\Phi_{3\times 4}=\;\left[\begin{array}{cccccccccccc} \varepsilon_{1}^{-} & 0 & 0 & \Theta_{1} & 0 & 0 & \Theta_{1}^{\prime } & 0 & 0 & 0 & 0 & 0\\ 0 & {\bar{\varepsilon }}_{1} & \varphi_{1} & 0 & \Theta_{1} & 0 & 0 & \Theta_{1}^{\prime } & 0 & 0 & 0 & 0\\ 0 & \varphi_{1} & {\bar{\varepsilon }}_{1} & 0 & 0 & \Theta_{1} & 0 & 0 & \Theta_{1}^{\prime } & 0 & 0 & 0\\ \Theta_{1} & 0 & 0 & {\bar{\varepsilon }}_{2} & \varphi_{2} & 0 & \Theta_{2} & 0 & 0 & \Theta_{2}^{\prime } & 0 & 0\\ 0 & \Theta_{1} & 0 & \varphi_{2} & {\bar{\varepsilon }}_{2} & 0 & 0 & \Theta_{2} & 0 & 0 & \Theta_{2}^{\prime } & 0\\ 0 & 0 & \Theta_{1} & 0 & 0 & \varepsilon_{2}^{-} & 0 & 0 & \Theta_{2} & 0 & 0 & \Theta_{2}^{\prime }\\ \Theta_{1}^{\prime } & 0 & 0 & \Theta_{2} & 0 & 0 & \varepsilon_{3}^{-} & 0 & 0 & \Theta_{3} & 0 & 0\\ 0 & \Theta_{1}^{\prime } & 0 & 0 & \Theta_{2} & 0 & 0 & {\bar{\varepsilon }}_{3} & \varphi_{3} & 0 & \Theta_{3} & 0\\ 0 & 0 & \Theta_{1}^{\prime } & 0 & 0 & \Theta_{2} & 0 & \varphi_{3} & {\bar{\varepsilon }}_{3} & 0 & v & \Theta_{3}\\ 0 & 0 & 0 & \Theta_{2}^{\prime } & 0 & 0 & \Theta_{3} & 0 & 0 & {\bar{\varepsilon }}_{4} & \varphi_{4} & 0\\ 0 & 0 & 0 & 0 & \Theta_{2}^{\prime } & 0 & 0 & \Theta_{3} & 0 & \varphi_{4} & {\bar{\varepsilon }}_{4} & 0\\ 0 & 0 & 0 & 0 & 0 & \Theta_{2}^{\prime } & 0 & 0 & \Theta_{3} & 0 & 0 & \varepsilon_{4}^{-} \end{array}\right],$$

where ${\bar{\varepsilon }}_{l}=\varepsilon_{l}^{-}-\varphi_{l}$, being l=1, 2, 3 or 4,

(A66) $$\varepsilon_{l}^{-}=\left[\begin{array}{ccc} e_{x,x}(l) & 0 & e_{x,z}(l)\\ 0 & e_{y,y}(l) & 0\\ e_{x,z}(l) & 0 & e_{z,z}(l) \end{array}\right]\mathrm{and}\;\varphi_{l}=-\gamma \left[\begin{array}{ccc} 3 & 0 & 0\\ 0 & 2+\cos(2\varphi_{l}) & \sin(2\varphi_{l})\\ 0 & \sin(2\varphi_{l}) & 2-\cos(2\varphi_{l}) \end{array}\right]$$

with $e_{x,x}(l)=\gamma [8+8\cos^{2}\theta_{l-1}+8\cos^{2}\theta_{l}+\cos(\theta_{l-2}+\theta_{l-1})+\cos(\theta_{l}+\theta_{l+1})]/4$, $e_{y,y}(l)=\;13\gamma /2$, $e_{z,z}(l)=8\gamma -e_{x,x}(l)$, $e_{x,z}(l)=\gamma [4\sin(2\theta_{l-1})+4\sin(2\theta_{l})+\sin(\theta_{l-2}+\theta_{l-1})+\sin(\theta_{l}+\theta_{l+1})]/4$,

(A67) $$\Theta_{l}=-\gamma \left[\begin{array}{ccc} 2+\cos(2\theta_{l}) & 0 & \sin(2\theta_{l})\\ 0 & 3 & 0\\ \sin(2\theta_{l}) & 0 & 2-\cos(2\theta_{l}) \end{array}\right],\;\mathrm{and}\;{\Theta^{\prime }}_{l}=-\frac{\gamma }{4}\left[\begin{array}{ccc} \cos(\theta_{l}+\theta_{l+1}) & 0 & \sin(\theta_{l}+\theta_{l+1})\\ 0 & 1 & 0\\ \sin(\theta_{l}+\theta_{l+1}) & 0 & -\cos(\theta_{l}+\theta_{l+1}) \end{array}\right]$$

The unitary transformation of the independent channel method is

(A68) $$\Xi_{3\times 4}=\frac{1}{\sqrt{6}}\;\left[\begin{array}{cccccccccccc} I_{3}\sqrt{2} & 0 & 2I_{3} & 0 & 0 & 0 & 0 & 0 & 0 & 0 & 0 & 0\\ I_{3}\sqrt{2} & -I_{3}\sqrt{3} & -I_{3} & 0 & 0 & 0 & 0 & 0 & 0 & 0 & 0 & 0\\ I_{3}\sqrt{2} & I_{3}\sqrt{3} & -I_{3} & 0 & 0 & 0 & 0 & 0 & 0 & 0 & 0 & 0\\ 0 & 0 & 0 & I_{3}\sqrt{2} & -I_{3}\sqrt{3} & -I_{3} & 0 & 0 & 0 & 0 & 0 & 0\\ 0 & 0 & 0 & I_{3}\sqrt{2} & I_{3}\sqrt{3} & -I_{3} & 0 & 0 & 0 & 0 & 0 & 0\\ 0 & 0 & 0 & I_{3}\sqrt{2} & 0 & 2I_{3} & 0 & 0 & 0 & 0 & 0 & 0\\ 0 & 0 & 0 & 0 & 0 & 0 & I_{3}\sqrt{2} & 0 & 2I_{3} & 0 & 0 & 0\\ 0 & 0 & 0 & 0 & 0 & 0 & I_{3}\sqrt{2} & -I_{3}\sqrt{3} & -I_{3} & 0 & 0 & 0\\ 0 & 0 & 0 & 0 & 0 & 0 & I_{3}\sqrt{2} & I_{3}\sqrt{3} & -I_{3} & 0 & 0 & 0\\ 0 & 0 & 0 & 0 & 0 & 0 & 0 & 0 & 0 & I_{3}\sqrt{2} & -I_{3}\sqrt{3} & -I_{3}\\ 0 & 0 & 0 & 0 & 0 & 0 & 0 & 0 & 0 & I_{3}\sqrt{2} & I_{3}\sqrt{3} & -I_{3}\\ 0 & 0 & 0 & 0 & 0 & 0 & 0 & 0 & 0 & I_{3}\sqrt{2} & 0 & 2I_{3} \end{array}\right]$$

which applied to Equation (A65) as ${\overset{⌣}{\Phi }}_{3\times 4}=\Xi_{3\times 4}^{T}\Phi_{3\times 4}\Xi_{3\times 4}$ yields

(A69) $${\overset{⌣}{\Phi }}_{3\times 4}=\frac{1}{2}\;\left[\begin{array}{cccccccccccc} 2\varepsilon_{1}^{-} & 0 & 0 & 2\Theta_{1} & 0 & 0 & 2\Theta_{1}^{\prime } & 0 & 0 & 0 & 0 & 0\\ 0 & 2\varepsilon_{1}^{+} & 0 & 0 & -\Theta_{1} & \sqrt{3}\Theta_{1} & 0 & 2\Theta_{1}^{\prime } & 0 & 0 & 0 & 0\\ 0 & 0 & 2\varepsilon_{1}^{-} & 0 & -\sqrt{3}\Theta_{1} & -\Theta_{1} & 0 & 0 & 2\Theta_{1}^{\prime } & 0 & 0 & 0\\ 2\Theta_{1} & 0 & 0 & 2\varepsilon_{2}^{-} & 0 & 0 & 2\Theta_{2} & 0 & 0 & 2\Theta_{2}^{\prime } & 0 & 0\\ 0 & -\Theta_{1} & -\sqrt{3}\Theta_{1} & 0 & 2\varepsilon_{2}^{+} & 0 & 0 & -\Theta_{2} & -\sqrt{3}\Theta_{2} & 0 & 2\Theta_{2}^{\prime } & 0\\ 0 & \sqrt{3}\Theta_{1} & -\Theta_{1} & 0 & 0 & 2\varepsilon_{2}^{-} & 0 & \sqrt{3}\Theta_{2} & -\Theta_{2} & 0 & 0 & 2\Theta_{2}^{\prime }\\ 2\Theta_{1}^{\prime } & 0 & 0 & 2\Theta_{2} & 0 & 0 & 2\varepsilon_{3}^{-} & 0 & 0 & 2\Theta_{3} & 0 & 0\\ 0 & 2\Theta_{1}^{\prime } & 0 & 0 & -\Theta_{2} & \sqrt{3}\Theta_{2} & 0 & 2\varepsilon_{3}^{+} & 0 & 0 & -\Theta_{3} & \sqrt{3}\Theta_{3}\\ 0 & 0 & 2\Theta_{1}^{\prime } & 0 & -\sqrt{3}\Theta_{2} & -\Theta_{2} & 0 & 0 & 2\varepsilon_{3}^{-} & 0 & -\sqrt{3}\Theta_{3} & -\Theta_{3}\\ 0 & 0 & 0 & 2\Theta_{2}^{\prime } & 0 & 0 & 2\Theta_{3} & 0 & 0 & 2\varepsilon_{4}^{-} & 0 & 0\\ 0 & 0 & 0 & 0 & 2\Theta_{2}^{\prime } & 0 & 0 & -\Theta_{3} & -\sqrt{3}\Theta_{3} & 0 & 2\varepsilon_{4}^{+} & 0\\ 0 & 0 & 0 & 0 & 0 & 2\Theta_{2}^{\prime } & 0 & \sqrt{3}\Theta_{3} & -\Theta_{3} & 0 & 0 & 2\varepsilon_{4}^{-} \end{array}\right],$$

where $\varepsilon_{l}^{+}=\varepsilon_{l}^{-}-2\varphi_{l}$. Furthermore, we introduce a permutation matrix given by

(A70) $$\Pi_{3\times 4}=\;\left[\begin{array}{cccccccccccc} I_{3} & 0 & 0 & 0 & 0 & 0 & 0 & 0 & 0 & 0 & 0 & 0\\ 0 & 0 & 0 & 0 & I_{3} & 0 & 0 & 0 & 0 & 0 & 0 & 0\\ 0 & 0 & 0 & 0 & 0 & I_{3} & 0 & 0 & 0 & 0 & 0 & 0\\ 0 & I_{3} & 0 & 0 & 0 & 0 & 0 & 0 & 0 & 0 & 0 & 0\\ 0 & 0 & 0 & 0 & 0 & 0 & I_{3} & 0 & 0 & 0 & 0 & 0\\ 0 & 0 & 0 & 0 & 0 & 0 & 0 & I_{3} & 0 & 0 & 0 & 0\\ 0 & 0 & I_{3} & 0 & 0 & 0 & 0 & 0 & 0 & 0 & 0 & 0\\ 0 & 0 & 0 & 0 & 0 & 0 & 0 & 0 & I_{3} & 0 & 0 & 0\\ 0 & 0 & 0 & 0 & 0 & 0 & 0 & 0 & 0 & I_{3} & 0 & 0\\ 0 & 0 & 0 & I_{3} & 0 & 0 & 0 & 0 & 0 & 0 & 0 & 0\\ 0 & 0 & 0 & 0 & 0 & 0 & 0 & 0 & 0 & 0 & I_{3} & 0\\ 0 & 0 & 0 & 0 & 0 & 0 & 0 & 0 & 0 & 0 & 0 & I_{3} \end{array}\right],$$

which applied to Equation (A69) through ${\overset{⌢}{\Phi }}_{3\times 4}=\Pi_{3\times 4}^{T}{\overset{⌣}{\Phi }}_{3\times 4}\Pi_{3\times 4}$ we obtain

(A71) $${\overset{⌢}{\Phi }}_{3\times 4}=\frac{1}{2}\left[\begin{array}{cccccccccccc} 2\varepsilon_{1}^{-} & 2\Theta_{1} & 2\Theta_{1}^{\prime } & 0 & 0 & 0 & 0 & 0 & 0 & 0 & 0 & 0\\ 2\Theta_{1} & 2\varepsilon_{2}^{-} & 2\Theta_{2} & 2\Theta_{2}^{\prime } & 0 & 0 & 0 & 0 & 0 & 0 & 0 & 0\\ 2\Theta_{1}^{\prime } & 2\Theta_{2} & 2\varepsilon_{3}^{-} & 2\Theta_{3} & 0 & 0 & 0 & 0 & 0 & 0 & 0 & 0\\ 0 & 2\Theta_{2}^{\prime } & 2\Theta_{3} & 2\varepsilon_{4}^{-} & 0 & 0 & 0 & 0 & 0 & 0 & 0 & 0\\ 0 & 0 & 0 & 0 & 2\varepsilon_{1}^{+} & 0 & -\Theta_{1} & \sqrt{3}\Theta_{1} & 2\Theta_{1}^{\prime } & 0 & 0 & 0\\ 0 & 0 & 0 & 0 & 0 & 2\varepsilon_{1}^{-} & -\sqrt{3}\Theta_{1} & -\Theta_{1} & 0 & 2\Theta_{1}^{\prime } & 0 & 0\\ 0 & 0 & 0 & 0 & -\Theta_{1} & -\sqrt{3}\Theta_{1} & 2\varepsilon_{2}^{+} & 0 & -\Theta_{2} & -\sqrt{3}\Theta_{2} & 2\Theta_{2}^{\prime } & 0\\ 0 & 0 & 0 & 0 & \sqrt{3}\Theta_{1} & -\Theta_{1} & 0 & 2\varepsilon_{2}^{-} & \sqrt{3}\Theta_{2} & -\Theta_{2} & 0 & 2\Theta_{2}^{\prime }\\ 0 & 0 & 0 & 0 & 2\Theta_{1}^{\prime } & 0 & -\Theta_{2} & \sqrt{3}\Theta_{2} & 2\varepsilon_{3}^{+} & 0 & -\Theta_{3} & \sqrt{3}\Theta_{3}\\ 0 & 0 & 0 & 0 & 0 & 2\Theta_{1}^{\prime } & -\sqrt{3}\Theta_{2} & -\Theta_{2} & 0 & 2\varepsilon_{3}^{-} & -\sqrt{3}\Theta_{3} & -\Theta_{3}\\ 0 & 0 & 0 & 0 & 0 & 0 & 2\Theta_{2}^{\prime } & 0 & -\Theta_{3} & -\sqrt{3}\Theta_{3} & 2\varepsilon_{4}^{+} & 0\\ 0 & 0 & 0 & 0 & 0 & 0 & 0 & 2\Theta_{2}^{\prime } & \sqrt{3}\Theta_{3} & -\Theta_{3} & 0 & 2\varepsilon_{4}^{-} \end{array}\right],$$

where the single and dual channels are, respectively, described by 4 × 4 (red color) and 8 × 8 (blue color) independent block matrices. Hence, the final unitary transformation in the independent channel method is $\Xi_{3\times 4}\Pi_{3\times 4}$, which maps a thin graphene ribbon with buckling and rippling corrugations onto decoupled single and dual channels, as illustrated in Equation (A71). Given that both $\Xi_{3\times 4}$ and $\Pi_{3\times 4}$ are independent of the corrugation angles $\theta_{l}$ and $\varphi_{l}$, the independent channel method can be applied to both the system and the leads, even when they exhibit different rippling and buckling disorders.

For a small corrugated graphene ribbon of width W=3 and length L=4 connected to two semi-infinite corrugated leads of the same width, the phonon transmittance (T) can be written as

(A72) $$T(\omega )=\sum_{j=0}^{N}T^{\left(j\right)}(\omega ),$$

where *N* is the number of dual channels (here N =1), and $T^{\left(j\right)}(\omega )$ is the phonon transmittance of the *j*-th channel given by

(A73) $$T^{\left(j\right)}(\omega )=\mathrm{Tr}\{[t^{\left(j\right)}(\omega ){]}^{†}t^{\left(j\right)}(\omega )\}$$

being $t^{\left(0\right)}$ a 6 × 6 transmission-amplitude matrix for the single channel and $t^{\left(j\right)}$ a 12 × 12 matrix for j≠0. Using Equations (A34) and (A36), the double-line transfer matrices $T_{j,n}$ take the form

(A74) $$T_{0,n}=\left[\begin{array}{cc} {\left(S_{n}^{\mathrm{inter}}\right)}^{-1}(\omega^{2}-S_{n}^{\mathrm{intra}}) & -{\left(S_{n}^{\mathrm{inter}}\right)}^{-1}{\left(S_{n-1}^{\mathrm{inter}}\right)}^{T}\\ I_{6} & 0 \end{array}\right]$$

for the single channel, and

(A75) $$T_{j,n}=\left[\begin{array}{cc} {\left(D_{j,n}^{\mathrm{inter}}\right)}^{-1}(\omega^{2}-D_{j,n}^{\mathrm{intra}}) & -{\left(D_{j,n}^{\mathrm{inter}}\right)}^{-1}{\left(D_{j,n-1}^{\mathrm{inter}}\right)}^{T}\\ I_{12} & 0 \end{array}\right]$$

for the *j*-th dual channel, where $I_{6}$ and $I_{12}$ denote the 6 × 6 and 12 × 12 identity matrices, respectively.

It is important to note that the three vibrational modes υ=x,y or z are not independent in corrugated graphene ribbons, and they should be studied in a correlated way, which produces larger transfer matrices as given in Equations (A74) and (A75). Extending Equation (A55), the elements of the transmission matrix $t^{\left(j\right)}(\omega )$ are

(A76) $$t_{m,s}^{\left(j\right)}=\sqrt{v_{j,m}/v_{j,s}}\;{\overset{⌢}{t}}_{m,s}^{\left(j\right)},$$

where $v_{j,s}$ is the group velocity of the *s*-th vibrational mode in the *j*-th channel of a corrugated semi-infinite periodic lead. Analogous to Equation (A55), ${\overset{⌢}{t}}^{\left(j\right)}$ is given by

(A77) $${\overset{⌢}{t}}^{\left(j\right)}=p_{+,+}^{\left(j\right)}-p_{+,-}^{\left(j\right)}{\left(p_{-,-}^{\left(j\right)}\right)}^{-1}p_{-,+}^{\left(j\right)},$$

where $p_{\pm ,\pm }^{\left(j\right)}$ and $p_{\pm ,\mp }^{\left(j\right)}$ are submatrices of the *j*-th channel transfer matrix $P^{\left(j\right)}={\left(Q_{j}^{\mathrm{Lead}}\right)}^{-1}M^{\left(j\right)}Q_{j}^{\mathrm{Lead}}$ that describes the phonon propagation from the left lead, through the system, and into the right lead. Here, $M^{\left(j\right)}=\prod_{n=0}^{L/2}T_{n}^{\left(j\right)}$ denotes the system transfer matrix, and $Q_{j}^{\mathrm{Lead}}$ is built by the eigenvectors, in the same way as for the single channel case in Equation (A41) and the dual channel case after Equation (A53).

To calculate $v_{j,s}$ and $Q_{j}^{\mathrm{Lead}}$ in rippled leads with a four-line period, as shown in Figure 2a,b”, the dynamical matrix of the single channel can be written in the reciprocal space as

(A78) $$S_{0}^{\mathrm{Lead}}(k)=\left[\begin{array}{cccc} \varepsilon^{-}(-\theta_{0}) & \Theta (-\theta_{0}) & {\Theta^{\prime }}_{+}(k,\theta_{0}) & \Theta (0)e^{-i4ka}\\ \Theta (-\theta_{0}) & \varepsilon^{-}(-\theta_{0}) & \Theta (0) & {\Theta^{\prime }}_{+}(k,-\theta_{0})\\ {\left[{\Theta^{\prime }}_{+}(k,\theta_{0})\right]}^{†} & \Theta (0) & \varepsilon^{-}(\theta_{0}) & \Theta (\theta_{0})\\ \Theta (0)e^{i4ka} & {\left[{\Theta^{\prime }}_{+}(k,-\theta_{0})\right]}^{†} & \Theta (\theta_{0}) & \varepsilon^{-}(\theta_{0}) \end{array}\right],$$

where $\Theta (\theta_{0})=-\gamma \left[\begin{array}{ccc} 2+\cos(2\theta_{0}) & 0 & \sin(2\theta_{0})\\ 0 & 3 & 0\\ \sin(2\theta_{0}) & 0 & 2-\cos(2\theta_{0}) \end{array}\right],$ $\Theta^{\prime }(\theta_{0})=-\frac{\gamma }{4}\left[\begin{array}{ccc} \cos\theta_{0} & 0 & \sin\theta_{0}\\ 0 & 1 & 0\\ \sin\theta_{0} & 0 & -\cos\theta_{0} \end{array}\right],$ and $\varepsilon^{-}(\theta_{0})=2\gamma \left[\begin{array}{ccc} 8+4\cos^{2}\theta_{0}+\cos\theta_{0} & 0 & 2\sin(2\theta_{0})\\ 0 & 13 & 0\\ 2\sin(2\theta_{0}) & 0 & 8-4\cos^{2}\theta_{0}-\cos\theta_{0} \end{array}\right]$ and ${\Theta^{\prime }}_{+}(k,\theta_{0})=\Theta^{\prime }(-\theta_{0})+\Theta^{\prime }(\theta_{0})e^{-i4ka}$.

The dynamical matrix of dual channels in the reciprocal space is

(A79) $$D_{j}^{\mathrm{Lead}}(k)=\left[\begin{array}{cccc} \varepsilon^{\mathrm{dual}}(-\theta_{0},-\varphi_{0}) & \Theta^{\mathrm{dual}}(-\theta_{0}) & {\tilde{\Theta }}_{+}^{\mathrm{dual}}(k,\theta_{0}) & \Theta^{\mathrm{dual}}(0)e^{-i4ka}\\ \Theta^{\mathrm{dual}}(-\theta_{0}) & \varepsilon^{\mathrm{dual}}(-\theta_{0},\varphi_{0}) & \Theta^{\mathrm{dual}}(0) & {\tilde{\Theta }}_{+}^{\mathrm{dual}}(k,-\theta_{0})\\ {\left[{\tilde{\Theta }}_{+}^{\mathrm{dual}}(k,\theta_{0})\right]}^{†} & \Theta^{\mathrm{dual}}(0) & \varepsilon^{\mathrm{dual}}(\theta_{0},-\varphi_{0}) & \Theta^{\mathrm{dual}}(\theta_{0})\\ \Theta^{\mathrm{dual}}(0)e^{i4ka} & {\left[{\tilde{\Theta }}_{+}^{\mathrm{dual}}(k,-\theta_{0})\right]}^{†} & \Theta^{\mathrm{dual}}(\theta_{0}) & \varepsilon^{\mathrm{dual}}(\theta_{0},\varphi_{0}) \end{array}\right],$$

where $\varepsilon^{\mathrm{dual}}(\theta_{0},\varphi_{0})\;=\;\left[\begin{array}{cc} \varepsilon^{+}(\theta_{0},\varphi_{0}) & 0\\ 0 & \varepsilon^{-}(\theta_{0}) \end{array}\right],$ $\Theta_{j,l}^{\;\mathrm{dual}}(\theta_{0})\;=\;\left[\begin{array}{cc} c_{j} & {\left(-1\right)}^{l+1}s_{j}\\ {\left(-1\right)}^{l}s_{j} & c_{j} \end{array}\right]⊗\Theta (\theta_{0}),$ ${\tilde{\Theta }}^{\mathrm{dual}}(\theta_{0})\;=I_{2}⊗\Theta^{\prime }(\theta_{0}),$ and ${\tilde{\Theta }}_{+}^{\mathrm{dual}}(k,\theta_{0})={\tilde{\Theta }}^{\mathrm{dual}}(-\theta_{0})+{\tilde{\Theta }}^{\mathrm{dual}}(\theta_{0})e^{-i4ka}$. Here, $c_{j}$ and $s_{j}$ are defined in Equation (A14), and $\varepsilon^{+}(\theta_{0},\varphi_{0})\;=\varepsilon^{-}(\theta_{0})-2\varphi (\varphi_{0})$ with $\varphi (\varphi_{0})=-\gamma \left[\begin{array}{ccc} 3 & 0 & 0\\ 0 & 2+\cos(2\varphi_{0}) & \sin(2\varphi_{0})\\ 0 & \sin(2\varphi_{0}) & 2-\cos(2\varphi_{0}) \end{array}\right]$.

The equations of motion for the single and double channels with corrugations are, respectively,

(A80) $$S_{0}^{\mathrm{Lead}}(k)\;w_{0,s}^{\mathrm{Lead}}(k)=\omega_{0,s}^{2}(k)\;w_{0,s}^{\mathrm{Lead}}(k)\;andD_{j}^{\mathrm{Lead}}(k)\;w_{j,s}^{\mathrm{Lead}}(k)=\omega_{j,s}^{2}(k)\;w_{j,s}^{\mathrm{Lead}}(k).$$

Hence, the group velocity of *s*-th vibrational mode in the *j*-th channel is $v_{j,s}=d\omega_{j,s}/dk$. For the single channel, the matrix of eigenvectors is $Q_{0}^{\mathrm{Lead}}=[w_{0,\;1}^{\mathrm{Lead}}(k_{1})\;w_{0,\;2}^{\mathrm{Lead}}(k_{2})\;\cdots \;w_{0,\;12}^{\mathrm{Lead}}(k_{12})\;]$ and for the dual channels $Q_{j}^{\mathrm{Lead}}=[w_{j,\;1}^{\mathrm{Lead}}(k_{1})\;w_{j,\;2}^{\mathrm{Lead}}(k_{2})\;\cdots \;w_{j,\;24}^{\mathrm{Lead}}(k_{24})\;]$, where $w_{j,s}^{\mathrm{Lead}}$ are the normalized eigenvectors.

In Figure A5, the transmittances of (a) single channel, (b) the dual channel, and (c) the graphene ribbon, obtained from Equations (A72) and (A73), are plotted versus the vibrational frequency (ω) for both pristine (gray lines) and corrugated (violet lines) narrow graphene ribbons, whose structures are sketched in their respective insets. The used rippling angles in both leads are $\varphi_{0}=\theta_{0}=10{}^{\circ}$, while the buckling profile of the suspended region is characterized by two angles $\theta_{1}=11{}^{\circ}$ and $\theta_{2}=0{}^{\circ}$. For the pristine case, we take $\theta_{l}=\varphi_{l}=0$.

The normalized trace (red circles) from the Kubo–Greenwood formula in Equation (A64) is shown in Figure A5c for the corrugated graphene ribbon consisting of 3 × 4 carbon atoms (red circles). These traces were calculated without the independent channel method, by adding an imaginary part $\eta =10^{-5}\omega_{\alpha }^{2}$ to $\omega^{2}$, and using two periodic graphene leads of 1,500,000 atoms in each of them.

Observe in Figure A5c the excellent agreement between the phonon transmittance obtained from transfer matrices via the independent channel method and the normalized trace from the Kubo–Greenwood formula without using the independent channel transformation, even in the presence of corrugation. This agreement validates the reliability of the new independent channel method combined with the transfer-matrix method developed for dual channels.

## Appendix C. Thermal Contact Resistance

For a suspended graphene ribbon supported by a trench with platinum electrodes on a crystalline silicon block, as shown in Figure 2c, the interface between graphene leads and platinum contacts gives rise to a thermal contact resistance $R_{c}$ [13]. This $R_{c}$ can be estimated by analyzing the measured thermal resistance (*R*) as a function of the graphene-ribbon length ($L_{G}$) and temperature (*T*), as shown in Figure A6, whose data were obtained from Figure 1c of Ref. [13] and Figure 7b of its supplementary information.

In Figure A6a–f, the ballistic thermal resistance of graphene

(A81) $$R_{G}^{\mathrm{ballistic}}(T)=\frac{1}{A}\left(\frac{1}{4.4\times 10^{5}\;T^{1.68}}+\frac{1}{1.2\times 10^{10}}\right),$$

derived from the ballistic conductance of Ref. [12], is plotted as magenta dashed lines, using the effective cross-sectional area A=Wh=(1.5 μm)(0.335 nm). Observe that for ribbon lengths $L_{G}\geq 1\;\mu m$, the measured thermal resistance scales linearly with $L_{G}$. A minimal thermal resistance appears near $L_{G}\approx 300\;\mathrm{nm}$, consequently, the difference between this minimum and $R_{G}^{\mathrm{ballistic}}$ can be interpreted as the thermal contact resistance $R_{c}$. In fact, the phonon mean free path in suspended graphene, about 300 nm [12], is consistent with the experimental data presented in Figure A6.

Hence, this thermal contact resistance can be calculated by

(A82) $$R_{c}(T)=R(T,0.3\;\mu m)-\Delta R_{G}^{\mathrm{corrugated}}(T,0.3\;\mu m)-R_{G}^{\mathrm{ballistic}}(T),$$

where $\Delta R_{G}^{\mathrm{corrugated}}(T,0.3\;\mu m)=R_{G}^{\mathrm{corrugated}}(T,0.3\;\mu m)-R_{G}^{\mathrm{flat}}(T,0.3\;\mu m)$ accounts for the diffusive thermal resistance originating from structural corrugation along suspended graphene ribbons. The quantities $R_{G}^{\mathrm{corrugated}}$ and $R_{G}^{\mathrm{flat}}$ can be calculated from Equation (18) as

(A83) $$R_{G}={\left\{\frac{\hbar^{2}}{2\pi k_{B}T^{2}}\int_{0}^{\infty }d\omega \frac{\omega^{2}\exp(\hbar \omega /k_{B}T)}{{\left[\exp(\hbar \omega /k_{B}T)-1\right]}^{2}}\;T(\omega )\right\}}^{-1},$$

whose spectral transmittance T(ω) is taken from Equation (A63) for $R_{G}^{\mathrm{flat}}$ and from Equation (A72) for $R_{G}^{\mathrm{corrugated}}$.

In Figure A7, the thermal contact resistance obtained from Equation (A82) is plotted as a function of temperature (blue squares). The fitted analytical expression (open circles) is given by

(A84) $$R_{c}(T)\;=(3.784\times 10^{6}K\;W^{-1})\left[{\left(\frac{T_{0}}{T}\right)}^{\beta }+\left(\frac{T}{T_{0}}\right)\exp\left(-9.44\frac{T_{0}}{T}\right)\right],$$

where $T_{0}=100\;K$ and β=1.274.

In Equation (A84), the first term represents the diffusive thermal resistance, whereas the second term arises from anharmonic Umklapp processes [42]. Note that the thermal contact resistance given by Equation (A84) will be added to the calculated thermal resistance when the theoretical results are compared with experimental data.
